# UHPLC‐HRMS/MS–Based Metabolic Profiling and Quantification of Phytochemicals in Different Parts of *Coccinia grandis* (L.) Voigt

**DOI:** 10.1002/fsn3.70004

**Published:** 2025-02-10

**Authors:** In Young Lee, Doo‐Hee Lee, Ju Hong Park, Nami Joo

**Affiliations:** ^1^ Department of Convergence IT Engineering Pohang University of Science and Technology (POSTECH) Pohang Republic of Korea; ^2^ National Instrumentation Center for Environmental Management Seoul National University Seoul Republic of Korea; ^3^ Department of Food and Nutrition Sookmyung Women's University Seoul Republic of Korea

**Keywords:** *Coccinia grandis*, phytochemicals, polyphenol, secondary metabolites, UHPLC‐HRMS

## Abstract

*Coccinia grandis* (L.) Voigt ( *C. grandis* ), a member of the Cucurbitaceae family, is recognized for its phytochemicals that possess antioxidant and antidiabetic properties, along with a wide array of nutritional and health‐promoting benefits. However, a comprehensive investigation of the phytochemical profiles and biologically active constituents in different parts of *C. grandis* has not yet been reported. Therefore, this study aimed to evaluate the phytochemical constituents of three distinct parts of *C. grandis* (fruit, leaves, and stem) at the same growth stage. The phytochemicals in *C. grandis* were identified using UHPLC‐HRMS–based untargeted metabolomics, followed by a quantitative analysis of the primary metabolites. The qualitative analysis revealed 60 secondary metabolites, including phenolic compounds (6 hydroxybenzoic acids, 22 hydroxycinnamic acids, 2 coumarins, 1 flavanone, 1 flavanonol, 2 flavones, 22 flavonols, and 2 lignans) and triterpenes (2 cucurbitacins). Furthermore, nine plant hormones and 30 amino acids were successfully identified. The quantitative analysis of 32 types of secondary metabolites indicated that the leaves contained the highest total amounts of flavonoids (501.37 mg/100 g) and hydroxycinnamic acids (1148.23 mg/100 g). Additionally, the analysis of amino acids revealed a total of 20 types, with the leaf extract exhibiting the highest total amounts of both essential and nonessential amino acids, followed by the fruit and stem extracts. In conclusion, the analysis of the primary and secondary metabolite composition and content of various parts of *C. grandis* demonstrated that the leaf extract replace with had the greatest functionality, suggesting its potential utility in the development of health functional foods.

## Graphical abstract

The phytochemicals in different parts of *C. grandis* were identified using UHPLC‐HRMS–based untargeted metabolomics, followed by a quantitative analysis of the primary metabolites. The analysis of the primary and secondary metabolite composition and content of various parts of *C. grandis* demonstrated that the leaf extract exhibited the greatest functionality.

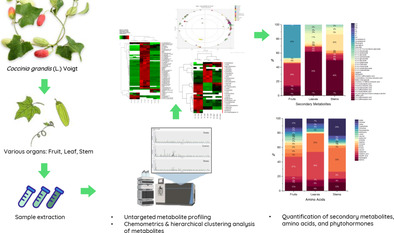

## 1 Introduction

Plant‐based medicinal substances have consistently provided substantial health benefits to humans throughout history. Their importance as a primary source of new bioactive compounds is due to their widespread availability, cost‐effectiveness, and profound cultural importance (Belwal et al. 2019; Checkouri et al. 2020; Gordon et al. 2012; Kiselova‐Kaneva et al. 2022; Kubola, Siriamornpun, and Meeso 2011). To investigate their natural antioxidant potential, extensive research has focused on medicinal and edible plants that contain these biologically active phytochemicals. Considerable quantities of bioactive compounds, such as phenolics, flavonoids, and tannins, have been identified in various plant organs, including leaves, fruits, stems, seeds, and roots, which are capable of inhibiting the excessive production of free radicals (Adebo and Gabriela Medina‐Meza 2020; Atanasov et al. 2015). Epidemiological and clinical investigations highlight that the majority of phytochemicals exhibit protective and disease‐preventing functions attributed to their antioxidant properties. These properties involve neutralizing lipid peroxides or preventing the breakdown of hydroperoxides into free radicals (Maisuthisakul, Suttajit, and Pongsawatmanit 2007; Unuofin, Otunola, and Afolayan 2018). Furthermore, synergistic mechanisms have been identified that result in more pronounced effects when a mixture of phytochemicals is present in fruits and vegetables, compared to individual phytochemicals (Isabelle et al. 2010). Consequently, studies that characterize and quantify phytochemicals have seen a noticeable increase in recent years.

*Coccinia grandis* (L.) Voigt ( *C. grandis* ), commonly known as ivy gourd or scarlet gourd, belongs to the Cucurbitaceae family (Shaina and Beevy 2012). *C. grandis* is native to the tropical zone of South East Asia, North, and Central Africa (Wasantwisut and Viriyapanich 2003). The cultivation in South Korea is a recent development attributed to climate change. *C. grandis* is a perennial climbing plant or creeper that climbs with axillary tendrils and can reach up to 20 m. Stems can grow to a maximum length of 5 m which are extensively branched. The size of leaves is approximately 5–10 cm in width and length with five triangulate lobes. The oval fruits are 3–6 cm long, and unripe fruits are green with pale spots, while ripe fruits are red (Holstein 2015). *C. grandis* fruits and leaves are noteworthy in traditional medicine for their broad nutritional and health‐promoting attributes, including antioxidant (Kondhare and Lade 2017; Lee and Joo 2022; Meenatchi, Purushothaman, and Maneemegalai 2017), antidiabetic (Chanda et al. 2020; Mohammed et al. 2016; Shree et al. 2021; Wasana et al. 2021), anti‐inflammatory (Albrahim et al. 2020; Banerjee, Mukherjee, and Maji 2022), anti‐adipogenic (Bunkrongcheap et al. 2014), and antidyslipidemic effects (Singh et al. 2007). These characteristics of *C. grandis* can be attributed to the extensive array of active biological substances present in its fruits and leaves, which are abundant in flavonoids, sterols, and triterpenes (Mukherjee et al. 2022a,b). In particular, previous studies have indicated that *C. grandis* fruits are most abundant in bioactive substances during the immature stage (Lee and Joo 2022). The type and concentration of compounds may vary depending on the stage of maturity, but they can also change based on the organs of the plant at the same developmental stage (Hazrati et al. 2022; Yakoub et al. 2018). Accordingly, several studies have evaluated bioactive components, which vary depending on various parts of plants (Chavan and Santa‐Catarina 2023; Choi et al. 2016; Tkacz et al. 2021; Yeasmen and Orsat 2023).

Untargeted profiling does not require any prior knowledge of potential compounds and can theoretically detect all analyzable compounds with the employed method. During the discovery phase, identifying as many metabolites related to biological processes as possible is essential. For untargeted ultra‐high performance liquid chromatography (UHPLC)‐MS–based metabolomics analyses, the primary MS platform is high‐resolution MS (HRMS), which can be utilized to acquire the exact mass and tandem MS (MS2) of metabolites. Quantification following untargeted metabolic profiling is typically employed in the verification phase to determine the content of specific metabolites. Selected reaction monitoring (SRM) with triple quadrupole mass spectrometry (TQMS) is the preferred method for quantification due to its high sensitivity, specificity, and excellent quantification capability (Lu, Bennett, and Rabinowitz 2008; Tsugawa et al. 2014). Advancements in TQMS technology, especially in terms of ionization efficiency and scanning rate, permit the simultaneous analysis of a large number of metabolites.

Thus, the present study used UHPLC‐HRMS–based untargeted metabolomics to investigate differences among organs of *C. grandis* . In addition, quantitative analysis of major metabolites was performed. This study utilized UHPLC‐HRMS–based untargeted metabolomics to compare the phytochemical profiles of different parts (fruit, leaf, and stem) of *C. grandis* at the same growth stage. It also included a quantitative analysis of key metabolites. To our knowledge, this is the first comprehensive investigation of the phytochemical composition and bioactive content in these plant parts, highlighting their potential for use in the nutraceutical industry. This study aims to evaluate the phytochemical composition of three different parts (fruit, leaf and stem) of *C. grandis* at the same growth stage to investigate their potential as a source of bioactive metabolites in the nutraceutical industries.

## 2 Materials and Methods

### 2.1 Reagents and Standards

LC/MS‐grade acetonitrile, methanol, and ether were purchased from Fisher Scientific (Pittsburgh, PA, USA). LC/MS‐grade formic acid, acetic acid, hydrochloric acid (HCl), and ammonium formate were purchased from Sigma‐Aldrich (St. Louis, MO, USA). For the derivatization of amino acids, a 3‐mM Fmoc reagent solution and a 0.4‐N potassium borate buffer were obtained from Agilent (Agilent Technologies, Palo Alto, CA, USA). Standards of hydroxycinnamic acids (3*‐O‐*caffeoylquinic acid, 4*‐O‐*caffeoylquinic acid, 5*‐O‐*caffeoylquinic acid, 4*‐O‐*coumaroylquinic acid, 3,4‐dihydroxycinnamic acid, 4*‐O‐*(Β‐D‐glucosyl)‐trans‐4‐sinapoyl alcohol, 3*‐O‐*feruloylquinic acid, 5*‐O‐*feruloylquinic acid, 4‐hydroxycinnamic acid, 4‐hydroxy‐3‐methoxycinnamic acid, 3*‐O‐*caffeoylquinic acid methyl ester, 3,5‐dimethoxy‐4‐hydroxycinnamic acid, and 4,5‐di*‐O‐*caffeoylquinic acid), flavanone (naringenin), flavone (apigenin), flavonols (quercetin (Q), Q‐3*‐O‐*robinobioside, ‐arabinoglucoside, ‐rhamnoside, rutinoside, galactoside, and kaempferol (K), K‐3*‐O‐*glucoside, ‐glucuronide, ‐sambubioside, ‐arabinoside, ‐rhamnoside, ‐(6″*‐O‐*p‐coumaroyl)‐glucoside, and K‐7*‐O‐*glucoside), lignan (pinoresinol), and phytohormones (abscisic acid, gibberellin A4, indol‐3‐acetic acid, jasmonic acid, and salicylic acid) were all purchased from Sigma‐Aldrich. Triterpenes (cucurbitacins) were purchased from Biofron (La Mirada, CA, USA).

### 2.2 Plant Materials

*C. grandis* were planted in March 2023 in the flat ground of Yongin‐si, Gyeonggi‐do, Korea at N 43°32′09″, E 13°33′01″. The cultivated ground consists of a mixture of sand and silt strata. The difference in soil temperature between summer and winter exceeds 5°C, and the average annual temperature is 13.3°C, classifying this area as part of the mesic habitat. As of 2023, the annual average humidity in the growing area was 69.5%, the total precipitation was 1409.4 mm, and the solar exposure rate was 52.67%. Plants were grown without fertilization and protection, and *C. grandis* fruits (immature, 12 ± 2 days after flower wither, firm in texture, bright green), leaves, and stems were manually harvested in August 2023. Plant material was collected during the period of mass flowering and fruiting, which is characterized by the maximum concentration of phenolic compounds in many higher plant species (Fernando, Abeysinghe, and Dharmadasa 2013; Ammar et al. 2012). Various parts from *C. grandis* were dried for at least 72 h using a freeze dryer (Ilshin Biobase, Dongducheon, Korea), ground with a cutter mill using a 6‐mm grid, and the powders were filtered through a 150‐μm mesh. Samples were stored in a deep freezer (New Brunswick Scientific Co., Buckinghamshire, England) at −80°C and used for analysis.

### 2.3 Untargeted Metabolite Profiling

Freeze‐dried *C. grandis* fruits, leaves, and stems were extracted with 80% aqueous methanol. Sample extract (1 μL) was injected into an UHPLC‐Orbitrap‐HRMS system (Thermo Fisher Scientific Inc., MA, USA) equipped with a CORTECS T3 column (2.1 mm × 150 mm × 1.6 μm particle size; 120 Å pore size, Waters Co., Milford, MA, USA). The column oven temperature was set at 45°C while maintaining a flow rate of 0.25 mL/min. Solvent A (0.1% formic acid in LC/MS‐grade water) and solvent B (0.1% formic acid acetonitrile in LC/MS‐grade acetonitrile) were prepared for the chromatographic separations. Seperation was conducted under the following gradient: 0–0.5 min, 3% B; 0.5–15.0 min, 3%–15% B; 15.0–50.0 min, 95% B; 50.0–55.0 min, 95% B; 55.0–56.0 min, 95%–5% B; 56.0–60.0 min, 5% B. The full‐scan monitoring was used and data‐dependent mass spectra (DDMS2) mode with the mass range set at 100–1500 m/z. Positive and negative ion modes were utilized to operate a Q‐Exactive Plus Orbitrap mass spectrometer (Thermo Fisher Scientific). A heated electrospray ionization (Heated‐ESI) source parameters were applied as follows: positive capillary voltage 3.5 kV and negative capillary voltage 3.0 kV; ion transfer tube and capillary temperature 350°C; sheath gas flow rate 46 arb; aux gas flow rate 11 arb; sweep gas flow rate 2 arb.

### 2.4 Quantification of Secondary Metabolites

Quantification of secondary metabolites was conducted by referring to a study by Lee and Joo (2022). Quantification of *C. grandis* extracts (80% methanol aqueous) was conducted using a Vanquish Flex UHPLC system (Thermo Fisher Scientific) equipped with a CORTECS C18 column (2.1 mm × 150 mm × 1.6 μm particle size; 120 Å pore size). The injection volume was 1 μL, and the column oven temperature and flow rate were set to be 45°C and 0.25 mL/min, respectively. Solvent A (0.1% formic acid in LC/MS‐grade acetonitrile) and solvent B (0.1% formic acid in LC/MS‐grade acetonitrile) were prepared for the chromatographic separations. Seperation was conducted under the following gradient: 0–0.5 min, 5% B; 0.5–1.0 min, 5%–25% B; 1.0–10.0 min, 100% B; 10.0–10.5 min, 100% B; 10.5–11.0 min, 100%–5% B; 11.0–15.0 min, 5% B. A Thermo TSQ Altis TQMS equipped with a Heated‐ESI II source operating polarity‐switching ion selected reaction monitoring (SRM) mode was utilized. The optimized retention time, ionization mode, adduct, precursor ion, and product ion of secondary metabolites in SRM mode are shown in Table S1. A Heated‐ESI source parameters were applied as follows: positive capillary voltage of 3.5 kV and negative capillary voltage of 2.5 kV; ion transfer tube and capillary temperature 320°C; sheath gas flow rate 50 arb; aux gas flow rate 10 arb; sweep gas flow rate 1 arb. Trace Finder 4.1 software was utilized for both data acquisition and processing.

### 2.5 Quantification of Phytohormones

Extracts were prepared according to a modified method described by Lee and Joo (2022). Samples (50 mg) with 0.8 mL of 0.1% HCl were sonicated for 30 min, and shaked for 30 min after adding 1 mL of methyl tertiary butyl ether (MTBE). The supernatant (0.8 mL) was collected and dried using a SpeedVac concentrator (Thermo Fisher Scientific) at 4°C. After collection, the pellet were suspended in 100 μL of 80% methanol aqueous solution. and analyzed using a UHPLC Vanquish system. Sample extract (3 μL) was injected into an UHPLC Vanquish system equipped with a CORTECS C18 column (2.1 mm × 150 mm × 2.7 μm particle size; 120 Å pore size, Waters Co.). The column oven temperature was set at 45°C while maintaining a flow rate of 0.3 mL/min. Solvent A (0.02% acetic acid in LC/MS‐grade water) and solvent B (0.02% acetic acid in LC/MS‐grade acetonitrile) were prepared for the chromatographic separations.

Seperation was conducted under the following gradient: 0–0.1 min, 5% B; 0.1–4.0 min, 5%–60% B; 4.0–4.3 min, 60%–90% B; 4.3–4.8 min, 90% B; 4.8–5.0 min, 90%–5% B; 5.0–7.0 min, 5% B. A Thermo TSQ Altis TQMS equipped with a Heated‐ESI II source operating polarity‐switching ion SRM mode was utilized. A Heated‐ESI source parameters were applied as follows: positive capillary voltage of 3.5 kV and negative capillary voltage of 2.5 kV; ion transfer tube and capillary temperature 320°C; sheath gas flow rate 50 arb; aux gas flow rate 10 arb; sweep gas flow rate 1 arb. Trace Finder 4.1 software was utilized for both data acquisition and processing.

### 2.6 Quantification of Free Amino Acids

Free amino acid content of *C. grandis* was analyzed by UHPLC‐MS/MS analysis after derivatization procedure (Horanni and Engelhardt 2013; Lee, Park, and Joo 2024). A 80% methanol extract of sample (100 μL) was mixed with 150 μL of 0.4 N potassium borate buffer and 400 uL of distilled water. Then, 150 μL of 3 mM Fmoc reagent solution was added and a vortex‐mixer and allowed to proceed at ambient temperature for 10 min. After adding 100 uL acetic acid (1 M), the mixture was filtered through a 0.20‐mm nylon membrane filter. Quantification of amino acid derivates was conducted using a Vanquish Flex UHPLC system equipped with a BEH C18 column (2.1 mm × 100 mm × 1.7 μm 130 Å pore size, Waters Co.). Solvent A (0.1% formic acid and 10 mM ammonium formate in LC/MS‐grade water) and solvent B (0.1% formic acid in LC/MS‐grade methanol) were prepared for the chromatographic separations. The injection volume was 1 μL, and the column oven temperature and flow rate were set to be 45°C and 0.4 mL/min, respectively. Seperation was conducted under the following gradient: 0–0.1 min, 5% B; 0.1–0.5 min, 5%–30% B; 0.5–6.0 min, 30%–100% B; 6.0–7.0 min, 100% B; 7.0–7.1 min, 100%–5% B; 7.1–10.0 min, 5% B. A Heated‐ESI source parameters were applied as follows: positive capillary voltage of 3.5 kV; ion transfer tube and capillary temperature 320°C; sheath gas flow rate 50 arb; aux gas flow rate 10 arb; sweep gas flow rate 1 arb. Trace Finder 4.1 software was utilized for both data acquisition and processing.

### 2.7 Statistical Analysis

For untargeted metabolite profiling, chromatographic data underwent processing with Compound Discoverer 3.3 (Thermo Fisher Scientific). Mass spectrum deconvolutions were carried out with the assistance of references of NIST (2017), HMD, and MoNA. Mean and standard deviation values are provided for the data, based on three separate determinations. Variance analysis was carried out using a one‐way analysis of variance (ANOVA). Differences between sample means were analyzed using Scheffe's test, with a significance threshold of 0.05. Principal component analysis (PCA) was achieved using SIMCA 18 (Umetrics, Umeå, Sweden).

## 3 Results and Discussion

### 3.1 Untargeted Metabolite Profiling of *C. grandis* in Various Parts

#### 3.1.1 Metabolite Profiles

Untargeted metabolites from *C. grandis* extracts obtained from various parts were analyzed using a UHPLC‐HRMS system. Unique fingerprints containing several diagnostic ions were observed in both positive and negative ion spectra (Figure S1). The rapid characterization of crude extracts was achieved through the UHPLC‐HRMS system's ability to generate chemical fingerprints, which has been applied in the identification of bioactive compounds, drugs, and other substances across a range of medicinal and horticultural plants.

Plants produce a diverse array of organic compounds, many of which appear unrelated to their growth and development. These compounds, known as secondary metabolites, are often unevenly distributed among specific taxonomic groups within the plant kingdom. Secondary metabolites play a crucial role in plant adaptation to their environments and serve as an important source of pharmaceuticals (Rao and Ravishankar 2002). The analysis identified 60 secondary metabolites, including phenolic compounds (6 hydroxybenzoic acids, 22 hydroxycinnamic acids, 2 coumarins, 1 flavanone, 1 flavanonol, 2 flavones, 22 flavonols, and 2 lignans) and triterpenes (2 cucurbitacins). Additionally, this study revealed the activation of several unique metabolites, such as abscisic acid and salicylic acid (both phytohormones), as well as amino acids that have been recognized as major functional components of *C. grandis* in previous research. A total of 11 phytohormones and 33 amino acids were successfully identified. The identification process utilized reference standards, along with UV spectra, MS fragmentation, and literature data (Ma et al. 2016; Pop et al. 2013; Tkacz et al. 2020). The mass spectrometric data of compounds in the methanol extract of *C. grandis* , categorized by their chemical family, are presented in Table 1.

**TABLE 1:** Metabolite profiles in various parts of *Coccinia grandis* .

#	Compound	RT (min)	Formula	Molecular weight	Precursor ion (m/z)	Error (ppm)	Adduct	Major fragment ions (m/z)	Peak area × 10^6^		
									Fruits	Leaves	Stems
Hydroxybenzoic acids and derivatives											
1	3,4,5‐trihydroxybenzoic acid	2.692	C_7_ H_6_ O_5_	170.0206	169.0133	−1.57	[M − H]^−^	125.0231, 169.0131, 97.0281, 69.0331, 81.0334	6.972	53.130	22.767
2	3,4‐dihydroxybenzoic acid	5.254	C_7_ H_6_ O_4_	154.0256	153.0182	−1.68	[M − H]^−^	108.9875, 64.9974, 109.0284, 152.9770, 80.0453	4.111	116.749	33.175
3	2,5‐dihydroxybenzoate 2*‐O‐*β‐D‐glucoside	7.149	C_13_ H_16_ O_9_	316.0797	315.0725	0.94	[M − H]^−^	56.7148, 73.3239, 65.3523, 116.3375, 57.7883	4.929	8.248	1.940
4	Protocatechuic aldehyde	7.642	C_7_ H_6_ O_3_	138.0305	137.0233	−1.40	[M − H]^−^	137.0234, 93.0331,136.0155, 109.0283, 108.8982	7.117	116.935	20.929
5	4‐hydroxybenzoic acid	8.253	C_7_ H_6_ O_3_	138.0306	137.0233	−1.26	[M − H]^−^	93.0332, 137.0233, 94.0366, 136.8912, 65.0382	14.347	10.900	63.187
6	3‐hydroxy‐4‐methoxybenzaldehyde	13.969	C_8_ H_8_ O_3_	152.0473	153.0547	−0.46	[M + H]^+^	111.0445, 125.0599, 65.0393, 93.03401, 153.0548	0.000	0.000	16.873
Hydroxycinnamic acids and derivatives											
7	5*‐O‐*caffeoylquinic acid	7.274	C_16_ H_18_ O_9_	354.0951	353.0878	0.12	[M − H]^−^	191.0555, 135.0440, 179.0338, 353.0871, 85.0281	352.228	2749.729	278.198
8	5*‐O‐*coumaroylquinic acid	9.712	C_16_ H_18_ O_8_	338.1003	337.0931	0.47	[M − H]^−^	119.04889, 163.0390, 191.0553, 337.0934, 93.0330	76.113	462.803	63.661
9	3*‐O‐*caffeoylquinic acid	10.834	C_16_ H_18_ O_9_	354.0949	353.0875	−0.62	[M − H]^−^	191.0555, 85.0280, 93.0330, 127.0388, 353.0885	626.110	10034.262	1485.701
10	3,4‐dihydroxycinnamic acid	11.439	C_9_ H_8_ O_4_	180.0413	179.0340	−1.24	[M − H]^−^	135.0440, 134.9870, 90.9967, 179.0338, 59.0126	44.672	202.165	21.296
11	4*‐O‐*(Β‐D‐glucosyl)‐trans‐4‐sinapoyl alcohol	11.492	C_17_ H_24_ O_9_	372.1421	373.1476	0.21	[M + H]^+^	395.1317, 232.0706, 233.0785, 185.0422, 396.1350	1.532	12.762	0.000
12	3*‐O‐*feruloylquinic acid	11.575	C_17_ H_20_ O_9_	368.1107	367.1033	−0.20	[M − H]^−^	134.0362, 193.0499, 117.0333, 367.1014, 149.0598	55.735	416.289	71.979
13	4*‐O‐*caffeoylquinic acid	11.736	C_16_ H_18_ O_9_	354.0951	353.0878	0.01	[M − H]^−^	135.0440, 173.0444, 191.0555, 179.03340, 93.0331	43.318	2291.831	163.951
14	1*‐O‐*caffeoylquinic acid	13.529	C_16_ H_18_ O_9_	354.0950	353.0877	−0.35	[M − H]^−^	191.0554, 85.0280, 161.02322, 127.03867, 353.0886	35.301	2190.149	91.802
15	3*‐O‐*coumaroylquinic acid	13.992	C_16_ H_18_ O_8_	338.1001	339.1073	−0.09	[M + H]^+^	191.0554, 93.0331, 119.0488, 163.0391, 87.0073	1087.717	1199.993	75.331
16	4*‐O‐*coumaroylquinic acid	14.381	C_16_ H_18_ O_8_	338.1004	337.0931	0.57	[M − H]^−^	173.0444, 93.0331, 191.0554, 119.0489, 163.0389	105.167	692.200	52.828
17	4‐hydroxycinnamic acid	15.338	C_9_ H_8_ O_3_	164.0465	163.0390	−1.12	[M − H]^−^	119.0490, 163.0389, 120.0522, 162.8919, 93.0332	144.255	102.020	96.477
18	5*‐O‐*feruloylquinic acid	15.796	C_17_ H_20_ O_9_	368.1106	369.1179	−0.25	[M + H]^+^	177.0547, 145.0286, 117.0338, 149.0598, 89.0391	35.018	445.817	41.795
19	2‐hydroxycinnamic acid	16.410	C_9_ H_8_ O_3_	164.0470	165.0546	−1.27	[M + H]^+^	147.04401, 119.0494, 91.0547, 165.0545, 148.0475	40.664	221.521	63.524
20	1*‐O‐*coumaroylquinic acid	16.430	C_16_ H_18_ O_8_	338.1001	337.0928	−0.26	[M − H]^−^	191.0555, 85.0280, 93.0331, 173.0451, 337.0939	485.694	2317.368	368.472
21	4‐hydroxy‐3‐methoxycinnamic acid	17.764	C_10_ H_10_ O_4_	194.0577	193.0500	−0.98	[M − H]^−^	134.0362, 178.0262, 90.9320, 193.0503, 149.0596	22.248	53.191	34.619
22	4*‐O‐*feruloylquinic acid	17.910	C_17_ H_20_ O_9_	368.1106	367.1033	−0.26	[M − H]^−^	191.0555, 85.0280, 93.0333, 367.1037, 134.0361	33.260	352.366	28.851
23	3*‐O‐*caffeoylquinic acid methyl ester	18.349	C_17_ H_20_ O_9_	368.1107	367.1034	−0.05	[M − H]^−^	93.0332, 134.0357, 367.1024, 173.0449, 87.0073	1.263	41.004	3.847
24	3,5‐dimethoxy‐4‐hydroxycinnamic acid	18.579	C_11_ H_12_ O_5_	224.0681	223.0606	−1.72	[M − H]^−^	193.0134, 208.0372, 149.0232, 223.0605, 164.0469	2.465	0.000	4.463
25	3,5‐di*‐O‐*caffeoylquinic acid	20.733	C_25_ H_24_ O_12_	516.1264	515.1191	−0.66	[M − H]^−^	173.0444, 179.0338, 135.0441, 191.0552, 515.1188	0.550	49.524	14.763
26	4,5‐di*‐O‐*caffeoylquinic acid	21.257	C_25_ H_24_ O_12_	516.1265	515.1191	−0.54	[M − H]^−^	191.0555, 179.0340, 135.0440, 353.0874, 284.0327	0.000	34.702	11.540
27	3,4‐di*‐O‐*caffeoylquinic acid	21.621	C_25_ H_24_ O_12_	516.1268	515.1195	0.00	[M − H]^−^	191.0550, 173.0447, 135.0440, 179.0339, 353.0913	0.000	4.634	1.725
28	Cinnamic acid	23.795	C_9_ H_8_ O_2_	148.0526	149.0599	0.95	[M + H]^+^	149.0235, 131.0494, 148.9771, 121.0286, 125.9612	2.701	15.923	11.067
Coumarins									0.000	0.000	0.000
29	7‐hydroxycoumarin	15.032	C_9_ H_6_ O_3_	162.0317	163.0390	−0.14	[M + H]^+^	139.9823, 116.9665, 163.0388, 135.0444, 80.9457	8.387	148.691	17.747
30	6‐methoxy‐7‐hydroxycoumarin	17.491	C_10_ H_8_ O_4_	192.0425	193.0499	1.17	[M + H]^+^	193.0499, 133.0289, 107.9603, 161.0597, 105.0704	0.000	10.118	9.210
Flavanone											
31	Naringenin	25.685	C_15_ H_12_ O_5_	272.0686	271.0613	0.28	[M − H]^−^	119.0490, 151.0025, 271.0613, 107.0126, 65.0019	0.000	0.000	12.505
Flavanonol							[M − H]^−^				
32	Taxifolin	19.014	C_15_ H_12_ O_7_	304.0585	303.0513	0.74	[M − H]^−^	174.1280, 158.0960, 91.9629, 303.1727, 96.9589	0.000	3.969	0.000
Flavones							[M − H]^−^				
33	Luteolin	24.176	C_15_ H_10_ O_6_	286.0479	285.0407	0.65	[M − H]^−^	285.0408, 133.0287, 151.0029, 199.0397, 214.1287	0.000	10.277	1.542
34	Apigenin	25.952	C_15_ H_10_ O_5_	270.0528	269.0456	−0.13	[M − H]^−^	269.0454, 117.0331, 149.0234, 225.0557, 151.0026	0.000	24.994	3.073
Flavonols											
35	Q‐3,7*‐O‐*diglucoside	17.282	C_27_ H_30_ O_17_	626.1492	625.1420	1.43	[M − H]^−^	300.0273, 301.0357, 463.0908, 246.262, 74.7765	0.527	6.733	0.992
36	Q‐3*‐O‐*rhamnoside‐7*‐O‐*glucoside	17.536	C_27_ H_30_ O_16_	610.1538	609.1467	0.73	[M − H]^−^	301.0349, 151.0028, 609.1188, 299.7224, 121.0283	0.000	15.408	1.645
37	Q‐3*‐O‐*robinobioside	18.591	C_27_ H_30_ O_16_	610.1536	611.1608	0.27	[M + H]^+^	303.0504, 611.2174, 85.0289, 465.1034, 71.0498	80.313	110.155	73.161
38	Q‐3*‐O‐*arabinoglucoside	18.986	C_26_ H_28_ O_16_	596.1383	595.1309	0.87	[M − H]^−^	300.0277, 271.0248, 595.1319, 255.0308, 301.0357	4.673	48.862	5.152
39	Q‐3*‐O‐*glucoside	19.356	C_21_ H_20_ O_12_	464.0954	465.1027	−0.09	[M + H]^+^	300.0276, 271.0247, 463.0884, 301.035, 255.0296	31.329	146.937	39.624
40	K‐3*‐O‐*glucoside‐7*‐O‐*rhamnoside	19.457	C_27_ H_30_ O_15_	594.1584	595.1656	−0.17	[M + H]^+^	287.0551, 85.029, 449.1082, 71.0498, 129.0548	22.157	217.740	27.637
41	Q‐3*‐O‐*rhamnoside	19.582	C_21_ H_20_ O_11_	448.1007	449.1080	0.36	[M + H]^+^	303.0501, 85.029, 71.0498, 129.0548, 229.0496	12.777	49.484	34.394
42	Q‐3*‐O‐*rutinoside	19.582	C_27_ H_30_ O_16_	610.1532	609.1459	−0.33	[M − H]^−^	300.0277, 271.0249, 609.1467, 301.0356, 255.03	1429.383	3877.443	3044.775
43	Q‐3*‐O‐*galactoside	19.935	C_21_ H_20_ O_12_	464.0953	463.088	−0.42	[M − H]^−^	300.0277, 271.0248, 301.0355, 463.0884, 255.0299	96.497	2346.096	577.093
44	K‐3*‐O‐*sambubioside	20.043	C_26_ H_28_ O_15_	580.1428	579.1355	0.01	[M − H]^−^	284.0328, 255.0297, 285.0404, 227.0347, 579.1359	8.215	480.727	12.578
45	Q‐3*‐O‐*arabinoside	20.651	C_20_ H_18_ O_11_	434.0850	433.0777	0.26	[M − H]^−^	300.0275, 271.0248, 255.0298, 433.0772, 301.0345	1.981	25.154	5.728
46	K‐3*‐O‐*glucoside	20.714	C_21_ H_20_ O_11_	448.1002	447.0930	−0.76	[M − H]^−^	255.0298, 284.0327, 227.0345, 447.0935, 285.0405	31.476	2456.597	181.954
47	K‐3*‐O‐*rutinoside	20.775	C_27_ H_30_ O_15_	594.1581	593.1510	−0.64	[M − H]^−^	287.0552, 449.108, 85.029, 71.0498, 129.0548	1387.264	8433.955	3633.934
48	K‐3*‐O‐*glucuronide	21.142	C_21_ H_18_ O_12_	462.0794	461.0721	−1.01	[M − H]^−^	285.0406, 229.0501, 461.0731, 257.0454, 85.028	124.860	846.697	57.000
49	K‐7*‐O‐*glucoside	21.154	C_21_ H_20_ O_11_	448.1002	447.0929	−0.85	[M − H]^−^	287.0552, 85.029, 97.0287, 69.0341, 153.0183	93.243	3636.562	673.038
50	Mearnsetin 3‐rhamnoside	21.453	C_22_ H_22_ O_12_	478.1116	479.1189	1.06	[M + H]^+^	317.0658, 302.0427, 85.0291, 69.0341, 153.0185	1.846	15.679	1.616
51	Dihydrokaempferol	21.517	C_15_ H_12_ O_6_	288.0636	287.0564	0.89	[M − H]^−^	125.0231, 259.0613, 287.0566, 177.0549, 151.0027	3.870	70.682	8.579
52	K‐3*‐O‐*arabinoside	21.553	C_20_ H_18_ O_10_	418.0903	419.0975	0.61	[M + H]^+^	255.0298, 284.0328, 227.0346, 417.0828, 285.0405	0.994	128.347	6.152
53	K‐3*‐O‐*rhamnoside	22.498	C_21_ H_20_ O_10_	432.1060	431.0986	0.70	[M − H]^−^	255.0295, 284.033, 227.035, 285.0397, 431.0968	27.906	5.004	1.543
54	K‐3*‐O‐*(6″*‐O‐p*‐coumaroyl)‐glucoside	24.157	C_30_ H_26_ O_13_	594.1372	593.1303	−0.2	[M − H]^−^	284.0317, 255.0306, 285.0413, 593.1298, 227.0346	0.763	0.000	0.595
55	Quercetin	24.167	C_15_ H_10_ O_7_	302.0429	301.0356	0.65	[M − H]^−^	151.0024, 301.0358, 107.0127, 121.028, 178.9978	5.636	45.300	5.110
56	Kaempferol	26.229	C_15_ H_10_ O_6_	286.0475	285.0403	−0.86	[M − H]^−^	285.0406, 229.0501, 93.0333, 185.0598, 239.0344	11.575	329.784	22.190
Lignans											
57	Syringaresinol O‐β‐D‐glucoside	21.208	C_28_ H_36_ O_13_	580.2160	579.2087	0.64	[M‐H]^−^	181.0496, 166.026, 417.1558, 387.1085, 402.1311	55.250	2.176	2.762
58	Pinoresinol	22.209	C_20_ H_22_ O_6_	358.1420	359.1493	0.93	[M + H]^+^	137.0599, 341.1381, 323.1278, 177.0909, 163.0751	3.832	0.000	0.000
Triterpenes											
59	Cucurbitacin D	27.128	C_30_ H_44_ O_7_	516.3086	561.3068	−0.29	[M + FA − H]^−^	561.3077, 165.0912, 59.0124, 479.2802, 424.224	573.343	0.000	0.000
60	Cucurbitacin B	31.046	C_32_ H_46_ O_8_	558.3193	603.3176	0.09	[M + FA − H]^−^	603.3165, 59.0126, 520.4935, 144.7399, 88.3391	7885.019	0.000	0.000
Phytohormones											
61	Gibberellin A24	16.287	C_20_ H_26_ O_5_	346.1782	347.1855	0.60	[M + H]^+^	347.1872, 301.1799, 329.1755, 273.1846, 229.159	13.317	0.000	0.000
62	Salicylic acid	18.663	C_7_ H_6_ O_3_	138.0305	137.0232	−1.51	[M − H]^−^	93.0332, 137.0233, 94.0364, 65.0382, 59.4029	10.578	218.483	185.466
63	Gibberellin A4	19.199	C_15_ H_24_ O_8_	332.1474	331.1401	0.91	[M − H]^−^	331.1552, 257.118, 243.1753, 213.1279, 269.1545	1.560	0.000	0.000
64	Gibberellin44	19.205	C_20_ H_28_ O_6_	364.1890	363.1816	1.04	[M − H]^−^	363.1812, 319.1911, 137.0958, 273.1868, 245.1904	42.003	0.000	0.000
65	Indole‐3‐acetic acid	20.507	C_10_ H_9_ N O_2_	175.0635	176.0707	0.67	[M + H]^+^	130.0651, 176.0704, 125.0025, 97.008, 106.9923	0.000	5.545	1.950
66	Gibberellin A36	21.716	C_20_ H_26_ O_6_	362.1734	361.1662	1.20	[M − H]^−^	361.2231, 73.0284, 199.1339, 197.1186, 87.044	1.906	0.000	0.000
67	Hydroxyjasmonic acid	22.862	C_12_ H_18_ O_4_	226.1204	227.1278	−0.35	[M + H]^+^	111.0445, 181.1222, 163.1119, 153.1274, 191.1072	0.000	17.174	9.311
68	Abscisic acid	23.513	C_15_ H_20_ O_4_	264.1362	263.1289	0.23	[M − H]^−^	219.1384, 151.0751, 204.1147, 138.0673, 201.128	4.283	19.894	17.962
69	Jasmonic acid	25.557	C_12_ H_18_ O_3_	210.1255	209.1179	−0.51	[M − H]^−^	147.1169, 81.0705, 119.0858, 105.0704, 91.0548	0.000	5.731	1.998
Amino acids											
70	L‐lysine	1.139	C_6_ H_14_ N_2_ O_2_	146.1056	147.1129	0.37	[M + H]^+^	84.0814, 130.0864, 72.0814, 73.0848, 147.1126	146.575	0.000	59.060
71	L‐glutamine	1.227	C_5_ H_10_ N_2_ O_3_	146.0687	147.0764	−1.20	[M + H]^+^	84.0449, 130.05, 101.0714, 102.0554, 147.0765	300.432	1805.936	133.936
72	L‐arginine	1.228	C_6_ H_14_ N_4_ O_2_	174.1117	175.1190	0.08	[M + H]^+^	70.0658, 175.1189, 60.0564, 116.0709, 130.0975	729.086	145.684	79.954
73	γ‐aminobutyric acid	1.238	C_5_ H_13_ N O	103.1000	104.1073	1.51	[M + H]^+^	104.1075, 60.0815, 58.0658, 59.0736, 86.097	6431.301	4634.612	4744.190
74	L‐threonine	1.243	C_4_ H_9_ N O_3_	119.0585	120.0658	1.06	[M + H]^+^	120.081, 74.0607, 103.0547, 56.0503, 102.0555	30.933	38.499	0.000
75	N,N‐dimethylarginine	1.264	C_8_ H_18_ N_4_ O_2_	202.1431	203.1504	0.68	[M + H]^+^	140.0709, 203.1506, 70.0658, 112.076, 130.0501	108.882	0.000	0.000
76	L‐glutamic acid	1.266	C_5_ H_9_ N O_4_	147.0528	146.0446	−1.46	[M − H]^−^	102.0547, 128.034, 146.0448, 59.0125, 85.028	95.162	67.791	19.648
77	L‐asparagine	1.273	C_4_ H_8_ N_2_ O_3_	132.0536	133.0609	0.66	[M + H]^+^	87.0268, 61.0114, 133.032, 85.029, 58.9958	0.000	143.962	63.329
78	L‐proline	1.283	C_5_ H_9_ N O_2_	115.0636	116.0708	2.08	[M + H]^+^	70.0656, 116.0705, 71.0687, 68.0501, 98.0598	1961.538	13069.346	9042.489
79	L‐pipecolic acid	1.306	C_6_ H_11_ N O_2_	129.0791	130.0863	0.60	[M + H]^+^	84.0813, 130.0863, 85.0846, 82.0657, 131.09	1674.772	921.836	2146.566
80	N‐acetylornithine	1.314	C_7_ H_14_ N_2_ O_3_	174.1005	175.1078	0.25	[M + H]^+^	70.0658, 112.0761, 116.071, 175.1076, 129.1025	128.112	69.699	0.000
81	L‐citrulline	1.329	C_6_ H_13_ N_3_ O_3_	175.0958	176.1031	0.65	[M + H]^+^	130.0864, 70.0658, 71.0498, 88.0763, 134.0815	64.611	0.000	0.000
82	N‐acetylvaline	1.343	C_7_ H_13_ N O_3_	159.0896	160.0969	0.30	[M + H]^+^	160.0968, 88.0762, 114.0916, 87.0446, 58.0659	1471.348	397.890	869.188
83	N‐acetylleucine	1.346	C_8_ H_15_ N O_3_	173.1053	174.1126	0.45	[M + H]^+^	174.1123, 128.0707, 156.1019, 87.0446, 82.0658	24.007	142.007	206.920
84	2‐hydroxyphenylalanine	1.420	C_9_ H_11_ N O_3_	181.0739	182.0812	−0.01	[M + H]^+^	136.0758, 123.0442, 165.0546, 119.0494, 91.0547	1596.254	105.348	345.071
85	N,N‐dihydroxyvaline	1.686	C_5_ H_11_ N O_4_	149.0702	150.0774	−9.62	[M + H]^+^	104.0533, 56.0503, 133.032, 61.0114, 102.0555	74.643	16.848	41.520
86	L‐valine	1.694	C_5_ H_11_ N O_2_	117.0793	118.0865	2.28	[M + H]^+^	72.0814, 55.055, 118.0865, 70.0658, 58.0659	614.240	758.918	604.440
87	L‐glutathione	1.701	C_10_ H_17_ N_3_ O_6_ S	307.0838	306.0765	0.10	[M − H]^−^	76.0222, 179.0484, 162.0219, 84.0449, 308.0912	18.935	0.000	0.000
88	L‐methionine	1.704	C_5_ H_11_ N O_2_ S	149.0511	150.0584	0.37	[M + H]^+^	150.0775, 104.0533, 56.0503, 61.0114, 133.0511	870.107	439.807	130.208
89	L‐leucine	1.750	C_6_ H_13_ N O_2_	131.0948	132.1021	1.61	[M + H]^+^	130.0861, 88.039, 85.0281, 131.0336, 73.028	1119.446	401.667	740.055
90	5‐oxo‐L‐proline	1.769	C_5_ H_7_ N O_3_	129.0428	130.0501	1.76	[M + H]^+^	84.0813, 70.0658, 130.0865, 112.0873, 74.0244	234.665	561.713	311.348
91	N‐acetylglutamic acid	1.880	C_7_ H_11_ N O_5_	189.0636	188.0558	−0.47	[M − H]^−^	102.0545, 128.034, 59.0125, 188.0929, 129.0186	16.850	7.540	7.243
92	L‐tyrosine	1.911	C_9_ H_11_ N O_3_	181.0741	182.0815	1.38	[M + H]^+^	136.0758, 123.0443, 165.0547, 119.0494, 147.0442	2318.889	213.499	565.032
93	L‐isoleucine	1.971	C_6_ H_13_ N O_2_	131.0933	130.0861	−9.87	[M − H]^−^	130.0861, 88.039, 85.0281, 130.0497, 73.028	30.347	10.070	25.721
94	L‐pyroglutamine	2.338	C_5_ H_8_ N_2_ O_2_	128.0588	129.0661	1.76	[M + H]^+^	84.0449, 130.05, 85.0289, 56.0502, 102.0555	16.830	4.112	0.000
95	L‐phenylalanine	3.490	C_9_ H_11_ N O_2_	165.0791	166.0865	0.84	[M + H]^+^	120.081, 103.0547, 131.0493, 166.0863, 107.0496	8459.428	18486.755	7744.257
96	2‐phenylethylamine	4.976	C_8_ H_11_ N	121.0894	122.0967	2.29	[M + H]^+^	105.0703, 122.0195, 97.0082, 79.0549, 103.0548	25.379	54.614	19.566
97	L‐tryptophan	6.831	C_11_ H_12_ N_2_ O_2_	204.0897	205.0970	−0.90	[M + H]^+^	188.0708, 146.0601, 118.0654, 144.0809, 159.0917	1950.069	12696.088	2687.913
98	N‐benzoylisoleucine	9.177	C_13_ H_17_ N O_3_	235.1210	236.1283	0.71	[M + H]^+^	190.1229, 69.0705, 213.924, 219.0687, 97.0653	0.000	8.587	8.243
99	N‐acetylserotonin	14.365	C_12_ H_14_ N_2_ O_2_	218.1057	219.1130	0.93	[M + H]^+^	160.0757, 202.0866, 147.0441, 86.0971, 194.9435	3.856	10.531	1.377

Abbreviations: K, kaempferol; Q, quercetin.

The most significant group of phenolic acids identified from the mass spectra of *C. grandis* includes hydroxycinnamic acid and its derivatives, such as 5*‐O‐*caffeoylquinic acid (m/z 353), 3*‐O‐*caffeoylquinic acid (m/z 353), 3*‐O‐*coumaroylquinic acid (m/z 339), and 1*‐O‐*coumaroylquinic acid (m/z 337). Additionally, within the flavonoid group, the flavonols identified as the most dominant include Q‐3*‐O‐*rutinoside (m/z 609), Q‐3*‐O‐*galactoside (m/z 463), K‐3*‐O‐*glucoside (m/z 447), K‐3*‐O‐*rutinoside (m/z 593), and K‐7*‐O‐*glucoside (m/z 447). Regarding amino acids, γ‐aminobutyric acid (m/z 104), L‐proline (m/z 116), L‐leucine (m/z 132), L‐tyrosine (m/z 182), L‐phenylalanine (m/z 166), and L‐tryptophan (m/z 205) were tentatively confirmed to be the most prevalent, exhibiting higher peak areas than the others.

#### 3.1.2 Chemometrics Analysis of Metabolites

The metabolites identified in various parts of *C. grandis* were subjected to chemometric analyses to investigate how variations could be identified and correlated with phytochemicals. A distinct separation among the fruits, leaves, and stems of *C. grandis* was evident in the PCA findings. In both positive and negative ionization modes, the PCA score plots accounted for a combined 95.4% of the total variance (Figure S2). The plots effectively illustrated a clear distinction between PC1 (72.5%) and PC2 (23.2%), with PC1 primarily driving the differentiation among the samples. According to PC1 in the loading plots, leaves exhibited higher quantities of flavonols (kaempferol, quercetin, isorhamnetin, K‐7‐rhamnoside, and tiliroside) and hydroxycinnamic acids (coumaroyl quinic acid, feruloylquinic acid, feruloyl hexoside, p‐coumaric acid glucoside, ferulic acid glucoside, and chlorogenic acid) compared to other parts (Figure S3). The high concentration of flavonoids in leaves is closely associated with the phenylpropanoid pathway, a major metabolic pathway that supports the synthesis of phenolic compounds. This pathway is actively upregulated in leaves to fulfill the demands of protection and physiological regulation (Pandey and Rizvi 2009). Flavonoids also play a crucial role in defense mechanisms that affect growth and development, as well as in plant signaling processes, including the regulation of oxidative stress. Consequently, they are particularly abundant in leaves, where they are essential for plant metabolism and survival. Additionally, flavonoids exhibit antioxidant properties by scavenging reactive oxygen species (ROS) generated during photosynthesis and in response to environmental stresses such as drought or pathogen attacks.

**FIGURE 1:**
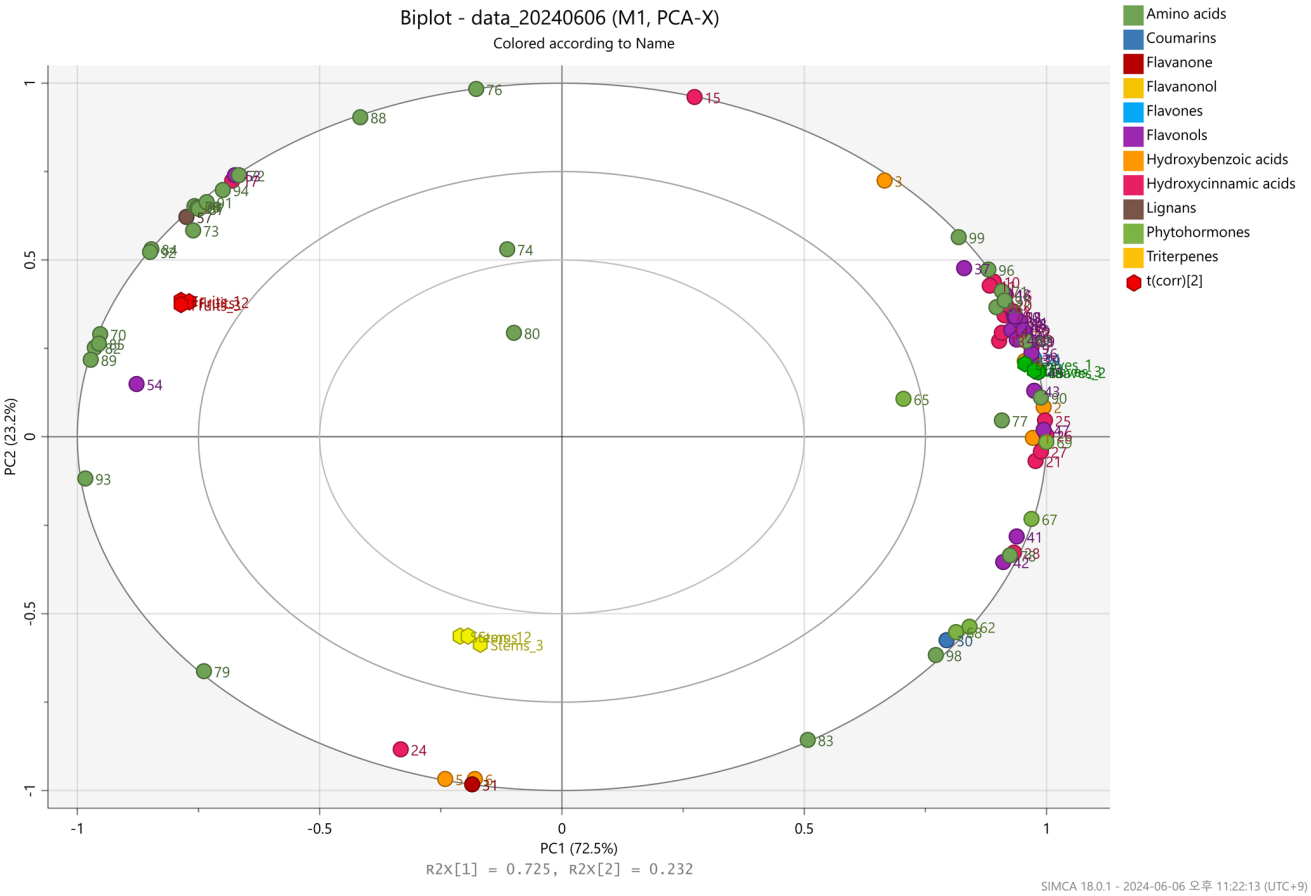
Principal component analysis (PCA) biplot obtained from metabolite profiling results for various parts of *Coccinia grandis*.

Conversely, most amino acids were found to be more prevalent in the fruits than in other parts. The biplot, which graphically depicts the PCA results, provides a visual understanding of marker distribution within *C. grandis* across its various parts (Figure 1). The biplot reveals that the distinctive distribution of flavonols and hydroxycinnamic acids in the second quadrant predominantly accounts for the variations in leaves compared to other parts. Additionally, primary amino acids (L‐leucine, L‐lysine, L‐tyrosine, 2‐hydroxyphenylalanine, and γ‐aminobutyric acid), as indicated by their position in the second quadrant of the biplot, contribute to explaining the variances observed in the fruits. These results suggest that most major bioactive compounds are likely to accumulate at higher concentrations in leaves than in fruits or stems.

#### 3.1.3 Hierarchical Clustering Analysis of Metabolites

**FIGURE 2:**
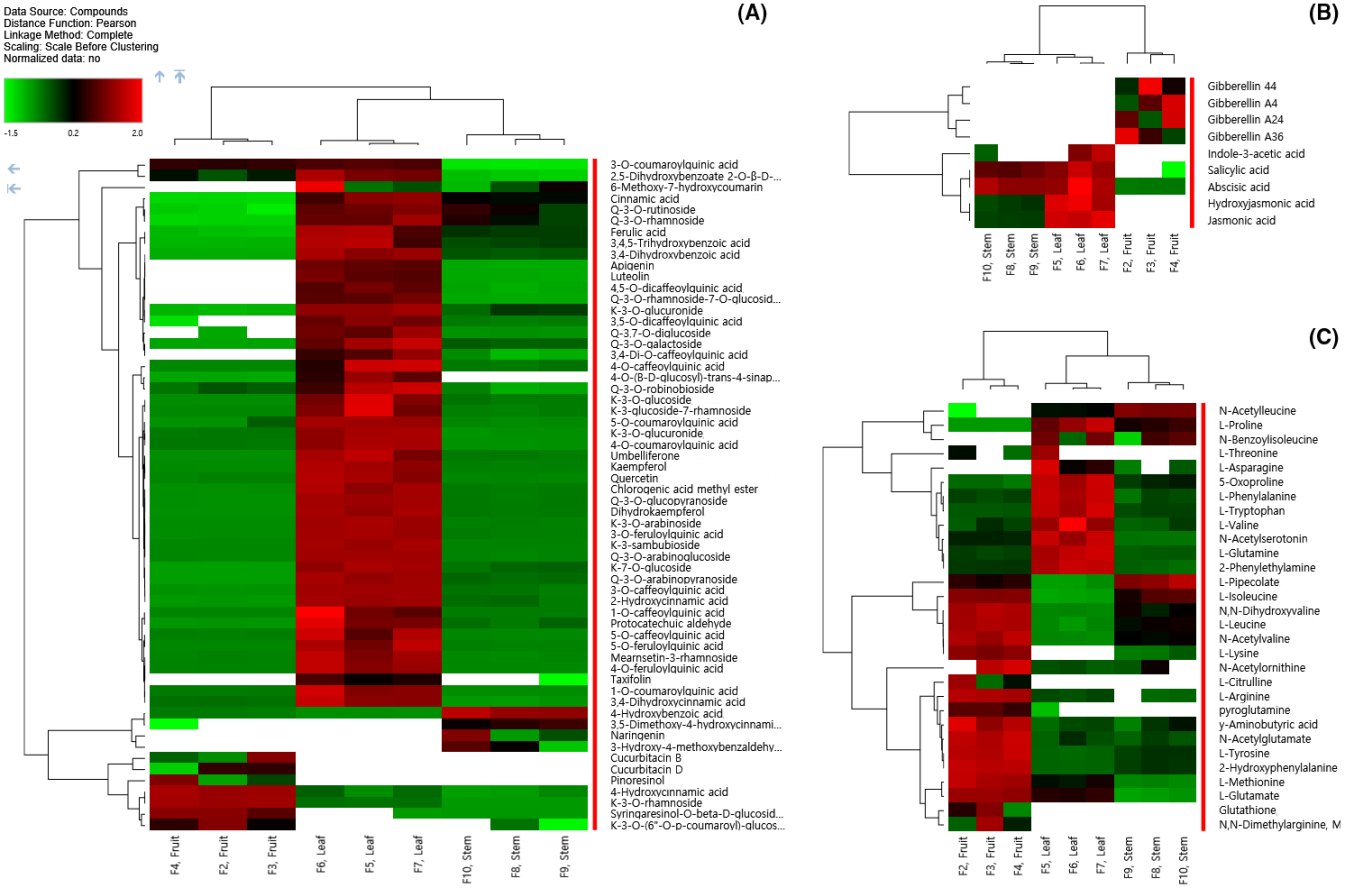
Heatmap of classified metabolites in various parts of *Coccinia grandis*. (A) Secondary metabolites, (B) phytohormones, (C) amino acid.

Hierarchical clustering analysis is useful for classifying metabolites with similar characteristics into groups and identifying trends in metabolite changes among experimental groups. Heatmap analysis indicated that the components of each category of differential metabolites (three classes) could be grouped into two clusters (I and II) (Figure 2). The identification of potential compounds contributing to the biological activity of *C. grandis* extracts involved performing a correlation analysis between samples and all identified metabolites, including phenolic compounds and free amino acids. As shown in Figure 2, several phenolic acids and flavonols identified as phenolic compounds exhibited strong correlations with the leaves. Flavonols (Q‐3*‐O‐*galactoside, Q‐3*‐O‐*robinobioside, K‐3*‐O‐*glucoside, and K‐3‐glucoside‐7‐rhamnoside) and hydroxycinnamic acids (4*‐O‐*caffeoylquinic acid, 1*‐O‐*caffeoylquinic acid, 5*‐O‐*caffeoylquinic acid, and 5*‐O‐*feruloylquinic acid) were the primary contributors. On the other hand, fruits and cucurbitacin B exhibited a moderate correlation. The stems demonstrated a similar correlation among hydroxycinnamic acids, cinnamic acid, and 3,5‐dimethoxy‐4‐hydroxycinnamic acid. This finding complements previous studies indicating that cucurbitacins B and D are abundant in the immature stages of *C. grandis* fruits. Of note, these hierarchical clustering results highlight distinct metabolic profiles across the leaves, fruits, and stems of *C. grandis* grown under identical conditions, reporting for the first time that each plant part plays a unique role in the plant's metabolic network. In particular, the significant differences between leaves and fruits suggest the presence of specialized metabolic pathways tailored to specific functions, such as defense and growth regulation. This indicates that the pharmacological effects of different parts of *C. grandis* may serve various purposes depending on the specific application.

### 3.2 Quantification of *C. grandis* in Various Parts

#### 3.2.1 Composition of Secondary Metabolites

Non‐targeted metabolite profiling using the UHPLC‐HRMS system revealed a high production of major phenolic compounds in the leaves of *C. grandis* . Therefore, major phytochemicals, including phenolic compounds, were selected for further analysis, and UHPLC‐MS/MS quantitative analysis was conducted. The selected major phytochemicals included hydroxycinnamic acids (5*‐O‐*caffeoylquinic acid, 3*‐O‐*caffeoylquinic acid, 3,4‐dihydroxycinnamic acid, 3*‐O‐*coumaroylquinic acid, 4*‐O‐*coumaroylquinic acid, 4‐hydroxycinnamic acid, 5*‐O‐*feruloylquinic acid, 4‐hydroxy‐3‐methoxycinnamic acid, and 4*‐O‐*feruloylquinic acid) and flavonols (Q‐3*‐O‐*rhamnoside, Q‐3*‐O‐*rutinoside, Q‐3*‐O‐*galactoside, K‐3*‐O‐*rutinoside, K‐7*‐O‐*glucoside, K‐3*‐O‐*rhamnoside, K‐3*‐O‐*(6″*‐O‐*p‐coumaroyl)‐glucoside, quercetin, and kaempferol), which have been quantified as secondary metabolites present in *C. grandis* in previous studies (Al‐Madhagy et al. 2019; Chanda et al. 2020; Lee, Park, and Joo 2024). Furthermore, the content of cucurbitacin, a triterpene with bioactive properties found in Cucurbitaceae plants (Ul Haq et al. 2019), was quantified. During the UHPLC separation of methanol extracts from different parts of *C. grandis* , the mobile phase was optimized using various aqueous phases and organic modifiers at different ratios. The optimized SRM mode successfully separated 30 phenolics, 2 triterpenes, 5 phytohormones, and 20 amino acids in the crude extract. These compounds were quantified using a standard calibration curve (Figures S4 and S5). The antioxidant effects of plant extracts are linked to the qualitative and quantitative presence of secondary metabolites, particularly phenolic compounds (Chaves, Santiago, and Alías 2020). This research represents the first report detailing the secondary metabolite composition of *C. grandis* extracts derived from various plant organs.

##### 3.2.1.1 Hydroxycinnamic Acids

UHPLC‐MS/MS analysis enabled the quantification of five caffeoylquinic acids, four hydroxycinnamic acids, two feruloylquinic acids, one coumaroylquinic acid, and one sinapoyl alcohol derivative (Table 2). Medicinal plants from the Cucurbitaceae family predominantly contain various hydroxycinnamic acids and their glycosides, including chlorogenic acid (3*‐O‐*caffeoylquinic acid), coumaric acid, and ferulic acid (Mukherjee et al. 2022b). Pharmacologically, these phenolic acids exhibit a variety of biological activities, including antioxidant, antimicrobial, antihepatotoxic, antiosteoporotic, antiulcer, immunomodulatory, and apoptotic effects (Gordon et al. 2012; Polbuppha et al. 2021; Adeyemi et al. 2014; Ye et al. 2015; Yoon et al. 2011). In this study, the average total content of hydroxycinnamic acids in *C. grandis* leaves was found to be 18 times more abundant than in its fruits and stems. Previous studies have demonstrated that the concentrations of hydroxybenzoic and hydroxycinnamic acid derivatives in the leaves are approximately five times higher than those in the fruit pulp of *H. rhamnoides* berries (Arimboor, Kumar, and Arumughan 2008). Additional reports have also indicated elevated levels of phenolic acids in the leaves of sea buckthorn (Tkacz et al. 2021).

**TABLE 2:** Quantification of secondary metabolites in various parts of *Coccinia grandis* .

#	Compound (mg/kg dm)	Fruits	Leaves	Stems	*F* value
Hydroxycinnamic acids					
1	5*‐O‐*caffeoylquinic acid	82.67 ± 4.15^b^	1220.79 ± 40.02^a^	69.25 ± 1.37 ^b^	2426.58***
2	3*‐O‐*caffeoylquinic acid	106.85 ± 0.57^c^	8999.13 ± 153.86^a^	399.44 ± 6.98^b^	9682.18***
3	3,4‐dihydroxycinnamic acid	1.78 ± 0.06^b^	8.50 ± 0.23^a^	1.00 ± 0.01^c^	2753.48***
4	4*‐O‐*(Β‐D‐glucosyl)‐trans‐4‐sinapoyl alcohol	20.30 ± 1.86^a^	4.16 ± 0.28^b^	0.37 ± 0.01^c^	282.45***
5	3*‐O‐*feruloylquinic acid	7.42 ± 0.16^c^	58.39 ± 1.79^a^	10.30 ± 0.15^b^	2265.17***
6	4*‐O‐*caffeoylquinic acid	2.90 ± 0.11^c^	298.77 ± 12.30^a^	14.45 ± 1.02^b^	1658.13***
7	4*‐O‐*coumaroylquinic acid	455.36 ± 22.42^b^	675.60 ± 35.39^a^	41.82 ± 2.08^c^	529.65***
8	4‐hydroxycinnamic acid	7.63 ± 0.11^a^	5.19 ± 0.09^b^	5.61 ± 0.35^b^	106.21***
9	5*‐O‐*feruloylquinic acid	8.98 ± 0.37^b^	180.59 ± 9.79^a^	9.67 ± 0.04^b^	916.39***
10	4‐hydroxy‐3‐methoxycinnamic acid	3.32 ± 0.17^c^	15.09 ± 1.72^a^	7.04 ± 0.18^b^	108.47***
11	3*‐O‐*caffeoylquinic acid methyl ester	0.00 ± 0.00^c^	0.50 ± 0.02^a^	0.10 ± 0.01^b^	1260.00***
12	3,5‐dimethoxy‐4‐hydroxycinnamic acid	0.55 ± 0.02^c^	0.95 ± 0.02^b^	1.61 ± 0.08^a^	350.87***
13	4,5‐di*‐O‐*caffeoylquinic acid	0.11 ± 0.01^c^	14.68 ± 0.36^a^	4.10 ± 0.13^b^	3642.52***
Flavanone					
14	Naringenin	0.01 ± 0.00^b^	2.91 ± 0.13^a^	0.09 ± 0.00^b^	1509.57***
Flavone					
15	Apigenin	nd	0.21 ± 0.01^a^	0.02 ± 0.00^b^	3748.00***
Flavonols					
16	Q‐3*‐O‐*robinobioside	10.39 ± 0.42^c^	94.86 ± 2.93^a^	18.89 ± 0.08^b^	2225.42***
17	Q‐3*‐O‐*arabinoglucoside	5.32 ± 0.12^c^	183.14 ± 4.32^a^	16.41 ± 0.36^b^	4744.55***
18	Q‐3*‐O‐*rhamnoside	1.07 ± 0.04^a^	0.00 ± 0.00^b^	0.00 ± 0.00^b^	2089.80***
19	Q‐3*‐O‐*rutinoside	57.56 ± 2.31^c^	357.76 ± 11.04^a^	212.88 ± 0.92^b^	1583.17***
20	Q‐3*‐O‐*galactoside	6.32 ± 0.20^c^	548.78 ± 6.72^a^	50.32 ± 0.46 ^b^	17979.27***
21	K‐3*‐O‐*glucoside	4.01 ± 0.25^b^	1868.47 ± 112.95^a^	36.45 ± 1.37^b^	803.28***
22	K‐3*‐O‐*glucuronide	14.96 ± 0.96^b^	480.30 ± 31.88^a^	7.55 ± 0.15^b^	648.88***
23	K‐3*‐O‐*sambubioside	2.07 ± 0.11^b^	454.62 ± 5.13^a^	8.63 ± 0.40^b^	22843.52***
24	K‐7*‐O‐*glucoside	2.16 ± 0.07^c^	968.51 ± 7.65^a^	19.11 ± 0.82^b^	4649.27***
25	K‐3*‐O‐*arabinoside	0.32 ± 0.01^c^	41.87 ± 1.37^a^	2.37 ± 0.12^b^	2607.47***
26	K‐3*‐O‐*rhamnoside	1.17 ± 0.03^a^	0.34 ± 0.00^b^	0.11 ± 0.01^c^	1948.00***
27	K‐3*‐O‐*(6″*‐O‐*p‐coumaroyl)‐glucoside	0.27 ± 0.01	nd	nd	—
28	Quercetin	0.18 ± 0.00^b^	0.93 ± 0.01^a^	0.06 ± 0.00^c^	11848.20***
29	Kaempferol	1.31 ± 0.09^b^	10.35 ± 0.27^a^	0.46 ± 0.09^c^	2957.09***
Lignan					
30	Pinoresinol	1.77 ± 0.04	nd	nd	—
Triterpenes					
31	Cucurbitacin B	684.89 ± 19.93^a^	nd	nd	—
32	Cucurbitacin D	16.04 ± 0.19	nd	nd	—

*Note:* Significant differences are represented by different superscripts within the column (*p* < 0.05); *** indicates significant effects at the level of *p* < 0.001.

Abbreviations: dm, dry matter; K, kaempferol; Q, quercetin.

3*‐O‐*caffeoylquinic acid, the predominant polyphenol in the human diet, is synthesized through the esterification of quinic acid and caffeic acid (Vázquez et al. 2012). In this study, it was quantitatively identified as the most dominant hydroxycinnamic acid. 3*‐O‐*caffeoylquinic acid was found to be the most abundant in leaves, with concentrations 80 times higher than those in fruits, which exhibited the lowest levels. Additionally, significant concentrations of other major hydroxycinnamic acids, such as 5*‐O‐*caffeoylquinic acid, 4*‐O‐*caffeoylquinic acid, 4*‐O‐*coumaroylquinic acid, and 5*‐O‐*feruloylquinic acid, were detected in leaves. Conversely, 4*‐O‐*coumaroylquinic acid in fruits and 3*‐O‐*caffeoylquinic acid in stems were identified as the most abundant hydroxycinnamic acids, highlighting significant variations depending on the specific parts of *C. grandis* . This variation arises because the plant contains not only free phenolic acids but also phenolic acids released from glycosidic bonds, which are prevalent in the fruit, as well as phenolic acids present as esters, which constitute the primary content of the leaves and stems. Understanding the accumulation and distribution of phenolic compounds in the various parts of *C. grandis* necessitates consideration of a wide range of factors. External environmental factors that influence specific growth patterns are one aspect. Internal factors, such as species‐specific accumulation of phenolic compounds and the timing of harvest, are also critical considerations. The impact of these elements, whether examined individually or in combination, can significantly affect the production of this group of secondary metabolites (Tungmunnithum et al. 2018; Dangles 2012). Consequently, variations in both qualitative and quantitative profiles can be attributed to the accumulation of phenolic acids, which is influenced by factors such as plant age, harvesting date, genetic and agronomic conditions, seasonal fluctuations, growth locations, and plant defense mechanisms (Fatima et al. 2015; Morgenstern et al. 2014). Furthermore, the detection of these compounds is affected by solvent polarity, extraction procedures, and chromatographic conditions (Arimboor, Kumar, and Arumughan 2008).

##### 3.2.1.2 Flavanone and Flavones

Naringenin, one of the flavanones identified in the profiling, and apigenin, one of the two flavones detected, were quantitatively analyzed. The leaves of *C. grandis* were found to be a superior source of naringenin (2.91 mg/kg) compared to the fruits and stems, which contained only trace amounts of naringenin at 0.01 mg/kg and 0.09 mg/kg, respectively. Naringenin (4′,5,7‐trihydroxyflavonone) is an aglycone derived from the hydrolysis of 7‐neohesperidoside (naringin) and naringenin 7‐rutinoside (narirutin) (Rani et al. 2016). This compound is primarily found in high concentrations in citrus fruits (Salehi et al. 2019) and is also present in the leaves and branches of non‐citrus plants (Díaz‐de Cerio et al. 2023; Lin et al. 2023). Apigenin (4′,5,7‐trihydroxyflavone) can be found in various plants either as an aglycone or in the form of several apigenin glycosides. *C. grandis* was found to contain trace amounts of apigenin, with 0.21 mg/kg in the leaves, 0.02 mg/kg in the stems, and 0.00 mg/kg in the fruits.

##### 3.2.1.3 Flavonols

Quantification using the UHPLC method indicated that *C. grandis* contained six quercetin and eight kaempferol derivatives (Table 2). Flavonols exhibited a tendency for glycosylation at Positions 3 and 7, forming structures such as *‐O‐*glucoside, *‐O‐*robinobioside, *‐O‐*rhamnoside, *‐O‐*rutinoside, *‐O‐*galactoside, *‐O‐*glucuronide, *‐O‐*sambubioside, and *‐O‐*arabinoside. Analysis of plant organs revealed greater variation in quercetin (Q) and kaempferol (K) derivatives in the leaves of *C. grandis* . The concentrations of flavonols can be arranged in the following order: fruits<stems < leaves. Each Q and K derivative exhibited significant differences depending on parts of *C. grandis* . Some important flavonols in *C. grandis* displayed low ionic intensity peaks in the stems; however, they had the highest concentrations in the leaves, including Q‐3*‐O‐*galactoside, K‐3*‐O‐*glucoside, K‐3*‐O‐*sambubioside, and K‐7*‐O‐*glucoside. Quercetin derivatives are widely recognized as the predominant polyphenolic fraction. The plant contains not only the aglycone quercetin but also various conjugated forms of quercetin, including glycosides and methyl ethers (Magar and Sohng 2020). In the present study, the leaves contained approximately six times more Q‐3*‐O‐*rutinoside (rutin) and 87 times more Q‐3*‐O‐*galactoside (hyperoside) than the fruits (Table 2). Similarly, the concentration of K derivatives was highest in the leaves. The content of K‐3*‐O‐*glucoside and K‐7*‐O‐*glucoside, the two predominant K derivatives in the leaves, was more than 400 times that found in the fruits. Lee and Joo (2022) quantified Q‐3*‐O‐*rutinoside as the main flavonol in immature fruits of *C. grandis* from Korea, reporting a content of 152.40 mg/kg, which was higher than the results of the present study. Kubola, Siriamornpun, and Meeso (2011) reported that immature fruits of *C. grandis* from Thailand contain 48.81 mg/g of Q‐3*‐O‐*rutinoside, while kaempferol was not detected.

Flavonoids, particularly flavonols, exhibit significant antioxidant properties and serve a protective function in a variety of pathological conditions, including inflammation, atherosclerosis, carcinogenesis, diabetes, and thrombosis [7–9]. Furthermore, flavonoids engage with several enzymatic systems to inhibit lipoxygenase and cyclooxygenase, thereby contributing to the prevention of cardiovascular diseases, exerting anti‐inflammatory effects, and facilitating cancer chemoprevention [13,14,16–18]. Quercetin and kaempferol derivatives have been recognized as the predominant flavonols found in various parts of *C. grandis* . Importantly, quercetin has demonstrated the potential to alleviate encephalomyelitis, an autoimmune disorder characterized by Th1 cell‐mediated immune responses [30]. The accumulation of flavonoids observed in plants may be linked to the enhanced activity of essential enzymes involved in the synthesis of phenolic compounds, such as phenylalanine ammonia‐lyase (PAL), as well as the metabolic pathways responsible for flavonoid biosynthesis and transformation (Gupta et al. 2011). However, the characteristic patterns of phenolic compounds present in *C. grandis* is not well understood due to the limited and fragmented experimental research data on this topic. Generally, the mesophyll, which is primarily concentrated in the leaves of plants, serves as a central tissue compartment for the biosynthesis of phenolic compounds (Goufo, Singh, and Cortez 2020; Rühmann et al. 2002). Additionally, it has been shown that the stem has a low mesophyll ratio, resulting in only a small amount of phenolic substances or their precursors being present, as these compounds are mobile (Gould et al. 2014). Consistent with the findings of previous studies, this research demonstrated the presence of redox‐active flavonoids and hydroxycinnamic acids in the leaves of *C. grandis* .

##### 3.2.1.4 Lignan

Among the various lignans, pinoresinol was quantified in different parts of *C. grandis* . The fruits of *C. grandis* exhibited the highest pinoresinol content, measuring 1.77 mg/kg, while pinoresinol was not detected in the stems and leaves. Lignans, as a category of dietary phytochemicals, have recently gained attention for their potential impact on human health (Adlercreutz 2007). As a dimer of coniferyl alcohol, pinoresinol is one of the simplest lignans and serves as a precursor for essential dietary lignans such as matairesinol and secoisolariciresinol (Adlercreutz 2007). Pinoresinol has demonstrated several biological effects, including anti‐inflammatory, antioxidant, and antiproliferative activities. A previous study reported that immature *C. grandis* fruits contained 2.99 mg/kg of pinoresinol, with its concentration increasing as the fruits matured (Lee and Joo 2022).

##### 3.2.1.5 Triterpenes

Based on the [M + FA‐H] ion, two cucurbitacins have been classified as triterpenes: cucurbitacin B and cucurbitacin D, both identified in *C. grandis* . Quantitative analysis revealed that these compounds were present in significant quantities exclusively in the fruit. The medicinal significance of plants in the Cucurbitaceae family is attributed to the presence of cucurbitacins, which are a class of triterpenes. Cucurbitacins B and D are the predominant types found within the Cucurbitaceae family (Miró 1995). Cucurbitacin B has demonstrated substantial antitumor activity and effectiveness in addressing inflammation and chronic hepatitis (Miró 1995). It has been shown to inhibit cell lines associated with lung, breast, and colon cancers. Furthermore, cucurbitacin D is recognized as a valuable antitumor agent that promotes apoptosis in human‐derived liver tumor cells (Takahashi et al. 2009) and possesses immunomodulatory properties that activate inflammasomes in macrophages (Song et al. 2013). The cucurbitacin content of *C. grandis* fruit investigated in this study was nearly consistent with data from previous research. Therefore, the immature fruit of *C. grandis* holds pharmacological potential and represents a promising source for functional foods and therapeutic compounds.

#### 3.2.2 Composition of Phytohormones

Phytohormones are increasingly recognized as alternatives to chemical substances for enhancing the nutritional value and improving the quality of agricultural products (Asghari 2019). The accumulation of phytohormones results from processed diets and the consumption of plant‐based sources. Occasionally, these plant hormones affect glucose metabolism, inflammation, antioxidant responses, cellular processes, cell division, cell cycle regulation, and cancer (Lin and Tan 2011). Consequently, elevated concentrations of these compounds in the human body are associated with various physiological and metabolic responses (Mukherjee et al. 2022a).

Five types of plant hormones—abscisic acid, gibberellin A4, indole‐3‐acetic acid, jasmonic acid, and salicylic acid—were quantitatively analyzed in this study (Table 3). These hormones were actively produced in the stems and immature fruits of *C. grandis* . Notably, salicylic acid was the most dominant plant hormone, present in substantial quantities in both fruits and stems, but absent in leaves. The protective effects of salicylic acid extend to shielding plants from various stressors, such as low temperatures, salinity, and water stress, while also participating in physiological processes ranging from seed germination to flowering and fruit ripening (Hadjipieri et al. 2020; Koo, Heo, and Choi 2020; Wei et al. 2011). Acting as a stress signal, salicylic acid initiates the accumulation of defensive molecules, which manifest as antioxidant compounds. Conversely, stems exhibited the highest concentration of abscisic acid, followed by fruits and leaves. Abscisic acid, a sesquiterpene, primarily regulates the initiation and maintenance of seed and shoot dormancy. In response to water stress, plants modify their membrane properties, with abscisic acid serving as a transcriptional activator (Pagare et al. 2015). Studies on the distribution of abscisic acid in plant organs have demonstrated that its concentration is typically highest in fruits, particularly during the ripening stage. This is attributed to abscisic acid's essential role in fruit development, ripening, and stress responses (Jia et al. 2011). In this study, a high concentration of abscisic acid was observed in the stem, which is believed to result from the harvesting of immature fruits at the growth stage rather than at the ripening stage. When humans regularly consume fruits and vegetables, abscisic acid can accumulate in the body. It has also been reported that abscisic acid regulates glucose homeostasis, as human adipose tissue releases this hormone at both high and low glucose levels (Zocchi et al. 2017). Additionally, gibberellins, which are involved in fruit and stem growth (Serrani et al. 2007), were found in small amounts exclusively in fruits. Jasmonic acid, a signaling molecule that regulates secondary metabolites and various phytochemicals, was found to have the highest concentration in leaves.

**TABLE 3:** Quantification of phytohormones in various parts of *Coccinia grandis* .

#	Compound (ng/kg dm)	Fruits	Leaves	Stems	*F* value
1	Abscisic acid	123.55 ± 4.50^b^	38.19 ± 0.43^c^	411.26 ± 5.52^a^	6759.01***
2	Gibberellin A4	21.61 ± 1.50^a^	nd	nd	—
3	Indol‐3‐acetic acid	12.23 ± 0.48^c^	30.83 ± 3.77^b^	77.16 ± 3.61^a^	366.21***
4	Jasmonic acid	10.93 ± 0.60^c^	92.30 ± 2.26^a^	62.33 ± 1.47^b^	1998.94***
5	Salicylic acid	1602.88 ± 36.60^a^	0.00 ± 0.00^c^	1308.12 ± 50.80^b^	1671.19***

*Note:* Significant differences are represented by different superscripts within the column (*p* < 0.05); *** indicates significant effects at the level of *p* < 0.001.

Abbreviation: dm, dry matter.

#### 3.2.3 Amino Acid Compositions

A total of 20 primary amino acids were quantitatively analyzed using UHPLC‐MS/MS. The composition and content of these amino acids in various parts of *C. grandis* are presented in Table 4. The free amino acids comprised nine nonessential amino acids, nine essential amino acids, and two non‐proteinogenic amino acids, with significant differences in content observed across each part of the plant. Among all the isolated amino acids, glutamine was found in remarkably high quantities, while lysine and methionine were identified as the limiting amino acids. The leaves contained the highest total amounts of both nonessential and essential amino acids, followed by the fruits and stems. The predominant essential amino acids in the leaves were tryptophan and phenylalanine, while arginine and leucine were most abundant in the fruits. The most prevalent nonessential amino acid in *C. grandis* by part was glutamine, followed by proline and alanine. Glutamine is the initial amino acid produced during nitrogen assimilation in plants (Lee, Liao, and Hsieh 2023). Glutamic acid is converted to glutamine by glutamine synthetase in plant leaves, and the resulting glutamine may serve as a precursor for asparagine (Kamachi et al. 1991). Glutamine and proline exhibited the highest concentrations in the leaves, whereas alanine content was slightly higher in the fruits. Many plants also contain unusual amino acids known as non‐protein amino acids, which can be integrated into proteins or exist in free forms, where they function as protective defensive substances. Non‐protein amino acids are distinct from the 22 proteinogenic amino acids that are naturally encoded in an organism's genome for protein synthesis. This study identified two non‐protein amino acids: citrulline and γ‐aminobutyric acid (GABA), with GABA being the most abundant in the stems of *C. grandis* .

**TABLE 4:** Quantification of amino acids in various parts of *Coccinia grandis* .

#	Compound (mg/100 g dm)	Fruits	Leaves	Stems	*F* value
Nonessential amino acids (NEAA)					
1	Alanine	139.08 ± 3.07^a^	132.89 ± 6.07^ab^	126.74 ± 1.75^b^	6.95***
2	Asparagine	16.07 ± 0.68^c^	169.68 ± 8.67^a^	46.83 ± 0.77^b^	780.44***
3	Aspartic acid	36.03 ± 1.09^a^	2.93 ± 0.21^c^	5.53 ± 0.10^b^	2471.57***
4	Glutamic acid	51.50 ± 1.52^a^	30.55 ± 1.27^b^	8.90 ± 0.30^c^	1013.28***
5	Glutamine	324.26 ± 7.16^b^	771.66 ± 33.40^a^	92.47 ± 0.73^c^	918.93***
6	Glycine	9.37 ± 0.36^a^	6.21 ± 0.61^c^	7.79 ± 0.21^b^	41.29***
7	Proline	54.57 ± 3.57^c^	513.76 ± 59.51^a^	345.44 ± 12.51^b^	130.90***
8	Serine	67.16 ± 1.88^a^	8.52 ± 0.70^c^	30.62 ± 0.49^b^	1853.98***
9	Tyrosine	66.79 ± 0.86^a^	2.83 ± 0.64^c^	7.18 ± 0.32^b^	9201.44***
Essential amino acids (EAA)					
10	Arginine	44.59 ± 0.92^a^	7.22 ± 0.27^b^	5.68 ± 0.24^c^	4490.79***
11	Histidine	10.43 ± 0.18^b^	18.34 ± 0.95^a^	6.58 ± 0.17^c^	335.23***
12	Leucine	64.56 ± 0.33^a^	17.61 ± 0.17^c^	27.32 ± 0.28^b^	18676.22***
13	Lysine	17.24 ± 0.50^a^	0.00 ± 0.00^c^	3.65 ± 0.07^b^	2956.10***
14	Methionine	14.49 ± 0.20^a^	6.85 ± 0.34^b^	1.30 ± 0.03^c^	2602.09***
15	Phenylalanine	29.63 ± 0.42^b^	77.52 ± 2.46 ^a^	25.68 ± 0.22^c^	1195.02***
16	Threonine	29.51 ± 0.74^a^	14.20 ± 0.58^c^	16.85 ± 0.20^b^	659.78***
17	Tryptophan	11.23 ± 0.60^b^	77.94 ± 3.51^a^	14.39 ± 0.63^b^	976.94***
18	Valine	41.57 ± 0.18^b^	57.42 ± 1.65 ^a^	39.04 ± 0.44^c^	303.89***
Non‐proteinogenic amino acids					
19	Citrulline	22.31 ± 0.46^a^	2.24 ± 0.14^c^	3.66 ± 0.02^b^	4830.83***
20	γ‐aminobutyric acid	149.18 ± 1.32^b^	136.03 ± 7.43^c^	250.74 ± 3.71^a^	501.90***

*Note:* Significant differences are represented by different superscripts within the column (*p* < 0.05); *** indicates significant effects at the level of *p* < 0.001.

Abbreviation: dm, dry matter.

## 4 Conclusions

This study investigated the metabolite profiles of *C. grandis* leaves, stems, and fruits for their potential use as functional food ingredients. Analysis using UHPLC‐HRMS–based untargeted metabolomics identified 60 secondary metabolites, including phenolic compounds (6 hydroxybenzoic acids, 22 hydroxycinnamic acids, 2 coumarins, 1 flavanone, 1 flavanonol, 2 flavones, 22 flavonols, and 2 lignans) and triterpenes (2 cucurbitacins). Additionally, nine plant hormones and 30 amino acids were successfully identified. Quantitative analysis of secondary metabolites indicated that leaves contained the highest total amounts and concentrations of flavonoids (flavanonol, flavones, and flavonols) and hydroxycinnamic acid. Cucurbitacins, the primary triterpenes found in *C. grandis* fruit, were not detected in the leaves or stems. Furthermore, quantification of a total of 20 amino acids revealed that leaves had the highest levels of both nonessential and essential amino acids. In contrast, abscisic acid and salicylic acid, which are significant plant hormones, exhibited the highest concentrations in stems and fruits, respectively. Among the various parts of *C. grandis* , leaves have the highest potential for producing food rich in antioxidants, such as phenolic compounds and amino acids. These findings provide valuable insights for adapting current technological methods to achieve the desired phytochemical profiles. Additionally, our findings pave the way for further investigation into the pharmaceutical uses of *C. grandis* and provide a foundation for refining cultivation and processing techniques to enhance the concentration of important bioactive compounds. Ultimately, *C. grandis* holds great promise as a natural source of health‐promoting metabolites, with potential applications in both functional foods and nutraceuticals.

## Author Contributions

**In Young Lee:** conceptualization (lead), data curation (lead), formal analysis (equal), investigation (lead), project administration (lead), resources (equal), software (lead), supervision (equal), visualization (lead), writing – original draft (lead). **Nami Joo:** writing – review and editing (equal). **Ju Hong Park:** funding acquisition (equal). **Doo‐Hee Lee:** methodology (equal), validation (equal).

## Ethics Statement

The authors have nothing to report.

## Conflicts of Interest

The authors declare no conflicts of interest.

## Supporting information


Data S1.

## Data Availability

The data that support the findings of this study are available from the corresponding author upon reasonable request.
