# Supplementary material for: UHPLC‐HRMS/MS–Based Metabolic Profiling and Quantification of Phytochemicals in Different Parts of Coccinia grandis (L.) Voigt
Source: Food Sci Nutr. 2025 Feb 10;13(2):e70004. doi: 10.1002/fsn3.70004 (PMC11808390; doi:10.1002/fsn3.70004)
Supplement: Supplementary file 1 — Data S1. [file FSN3-13-e70004-s001.docx]

Table S1. SRM parameters of metabolites

| **#** | Compound | Adduct | Precursor Ion (*m/z*) | Quantifier Ion (*m/z*) [CE (V)] | Qualifier Ion 1 (*m/z*) [CE (V)] | Qualifier Ion 2 (*m/z*) [CE (V)] |
| --- | --- | --- | --- | --- | --- | --- |
|  | ***Hydroxycinnamic acids*** |  |  |  |  |  |
| 1 | 5*-O-*Caffeoylquinic acid | [M−H]^−^ | 353.1 | 191.1[18.9] | 135.1[31.0] | 179.1[18.0] |
| 2 | 3*-O-*Caffeoylquinic acid | [M−H]^−^ | 353.1 | 191.1[16.4] | 85.1[40.1] | 179.1[15.0] |
| 3 | 3,4-Dihydroxycinnamic acid | [M−H]^−^ | 179.0 | 135.1[15.2] | 107.1[22.3] | 117.1[24.3] |
| 4 | 4*-O-*(Β-D-glucosyl)-trans-4-sinapoyl alcohol | [M+Na]^+^ | 395.2 | 232.1[24.5] | 185.1[20.9] | 233.1[20.4] |
| 5 | 3*-O-*Feruloylquinic acid | [M−H]^−^ | 367.1 | 193.1[16.6] | 134.1[31.8] | 179.1[21.6] |
| 6 | 4*-O-*Caffeoylquinic acid | [M−H]^−^ | 353.1 | 173.1[16.1] | 179.1[15.4] | 191.0[22.8] |
| 7 | 4*-O-*Coumaroylquinic acid | [M−H]^−^ | 337.1 | 93.1[34.8] | 163.1[16.8] | 173.1[14.8] |
| 8 | 4-Hydroxycinnamic acid | [M−H]^−^ | 163.0 | 119.1[14.6] | 93.1[30.7] | 117.1[32.3] |
| 9 | 5*-O-*Feruloylquinic acid | [M−H]^−^ | 367.1 | 191.1[15.8] | 173.1[18.1] | 193.1[19.8] |
| 10 | 4-Hydroxy-3-methoxycinnamic acid | [M−H]^−^ | 192.9 | 134.0[14.4] | 149.0[16.5] | 178.0[11.1] |
| 11 | 3*-O-*Caffeoylquinic acid methyl ester | [M−H]^−^ | 367.1 | 135.1[31.1] | 161.1[27.7] | 179.1[21.8] |
| 12 | 3,5-Dimethoxy-4-hydroxycinnamic acid | [M−H]^−^ | 222.9 | 208.0[13.9] | 164.1[15.9] | 193.0[21.9] |
| 13 | 4,5-Di*-O-*caffeoylquinic acid | [M−H]^−^ | 515.0 | 353.1[18.2] | 173.1[29.1] | 179.1[29.4] |
|  | ***Flavanone*** | [M−H]^−^ |  |  |  |  |
| 1 | Naringenin | [M−H]^−^ | 271.1 | 151.1[17.6] | 119.1[26.1] | 177.1[16.8] |
|  | ***Flavone*** | [M−H]^−^ |  |  |  |  |
| 1 | Apigenin | [M−H]^−^ | 269.1 | 117.1[35.0] | 149.1[24.3] | 151.1[24.5] |
|  | ***Flavonols*** |  |  |  |  |  |
| 1 | Q-3*-O-*robinobioside | [M−H]^−^ | 609.1 | 300.1[36.4] | 255.1[55.0] | 271.1[54.6] |
| 2 | Q-3*-O-*arabinoglucoside | [M−H]^−^ | 595.1 | 300.1.[35.9] | 271.1[55.0] | 301.1[30.8] |
| 3 | Q-3*-O-*rhamnoside | [M−H]^−^ | 447.1 | 300.1[25.1] | 271.1[42.5] | 301.1[21.9] |
| 4 | Q-3*-O-*rutinoside | [M−H]^−^ | 609.1 | 300.1[36.8] | 271.1[54.0] | 301.1[30.6] |
| 5 | Q-3*-O-*galactoside | [M−H]^−^ | 463.1 | 300.1[27.1] | 255.1[42.9] | 271.1[43.8] |
| 6 | K-3*-O-*glucoside | [M−H]^−^ | 447.1 | 284.1[27.7] | 227.1[47.0] | 255.1[41.1] |
| 7 | K-3*-O-*glucuronide | [M−H]^−^ | 461.1 | 285.1[19.4] | 229.1[37.2] | 257.1[32.6] |
| 8 | K-3*-O-*sambubioside | [M−H]^−^ | 579.1 | 284.1[31.6] | 227.1[54.7] | 55.1[51.8] |
| 9 | K-7*-O-*glucoside | [M−H]^−^ | 447.1 | 285.1[24.0] | 151.1[37.9] | 257.2[32.8] |
| 10 | K-3*-O-*arabinoside | [M−H]^−^ | 417.1 | 284.1[25.4] | 255.1[37.9] | 285.1[18.6] |
| 11 | K-3*-O-*rhamnoside | [M−H]^−^ | 431.1 | 285.1[19.8] | 255.1[39.2] | 284.1[25.6] |
| 12 | K-3*-O-*(6"*-O-*p-coumaroyl)-glucoside | [M−H]^−^ | 593.1 | 285.1[29.2] | 255.1[53.8] | 284.1[35.0] |
| 13 | Quercetin | [M−H]^−^ | 301.1 | 151.1[21.0] | 121.1[25.6] | 179.1[17.6] |
| 14 | Kaempferol | [M+H]^+^ | 287.2 | 153.0[33.6] | 121.1[32.7] | 165.1[28.9] |
|  | ***Lignan*** |  |  |  |  |  |
| 1 | Pinoresinol | [M+H]^+^ | 359.1 | 341.2[6.4] | 137.1[21.5] | 323.2[11.5] |
|  | ***Triterpenes*** |  |  |  |  |  |
| 1 | Cucurbitacin B | [M+FA-H]^−^ | 603.2 | 497.3[16.5] | 45.0[55.0] | 411.3[27.8] |
| 2 | Cucurbitacin D | [M+FA-H]^−^ | 561.2 | 515.3[16.0] | 45.1[55.0] | 497.3[18.4] |
|  | ***Phytohormones*** |  |  |  |  |  |
| 1 | Abscisic acid | [M−H]^−^ | 263.1 | 153.2[11.1] | 204.2[19.1] | 219.2[13.6] |
| 2 | Gibberellin A4 | [M−H]^−^ | 331.0 | 243.2[17.8] | 213.1[29.8] | 257.1[22.6] |
| 3 | Indol-3-acetic acid | [M+H]^+^ | 176.0 | 130.1[16.0] | 77.0[42.7] | 103.1[32.3] |
| 4 | Jasmonic acid | [M−H]^−^ | 209.0 | 59.0[12.8] | 41.1[40.9] | 165.2[12.3] |
| 5 | Salicylic Acid | [M−H]^−^ | 137.0 | 93.1[16.3] | 65.1[29.1] | 75.1[31.7] |
|  | ***Nonessential amino acids (NEAA)*** | |  |  |  |  |
| 1 | Alanine | [M+H]^+^ | 312.1 | 179.1[17.3] | 176.1[14.8] | 178.2[42.2] |
| 2 | Asparagine | [M+H]^+^ | 355.1 | 179.1[22.9] | 116.1[16.4] | 178.2[49.8] |
| 3 | Aspartic acid | [M+H]^+^ | 356.1 | 179.1[23.3] | 160.0[16.8] | 211.1[23.5] |
| 4 | Glutamic acid | [M+H]^+^ | 370.2 | 179.1[27.3] | 178.2[51.3] | 180.1[25.4] |
| 5 | Glutamine | [M+H]^+^ | 369.1 | 179.1[23.7] | 147.1[10.4] | 178.2[50.8] |
| 6 | Glycine | [M+Na]^+^ | 320.1 | 179.2[26.6] | 178.2[47.8] | 263.1[15.8] |
| 7 | Proline | [M+H]^+^ | 388.1 | 179.1[20.0] | 116.1[11.1] | 178.2[47.4] |
| 8 | Serine | [M+H]^+^ | 328.1 | 179.1[19.9] | 178.2[44.9] | 287.0[5.3] |
| 9 | Tyrosine | [M+H]^+^ | 404.1 | 179.1[21.9] | 178.2[55.0] | 404.1[8.9] |
|  | ***Essential amino acids (EAA)*** | [M+H]^+^ |  |  |  |  |
| 1 | Arginine | [M+H]^+^ | 397.2 | 179.1[30.2] | 158.1[21.4] | 175.2[23.3] |
| 2 | Histidine | [M+H]^+^ | 378.2 | 179.1[26.3] | 156.1[17.6] | 178.2[51.6] |
| 3 | Leucine/Isoleucine | [M+H]^+^ | 354.1 | 179.1[19.1] | 132.2[8.8] | 178.2[48.3] |
| 4 | Lysine | [M+H]^+^ | 369.2 | 179.1[25.4] | 130.1[18.8] | 178.2[52.4] |
| 5 | Methionine | [M+H]^+^ | 372.1 | 179.1[22.8] | 176.0[17.0] | 178.2[48.9] |
| 6 | Phenylalanine | [M+H]^+^ | 388.1 | 179.1[22.4] | 166.1[9.8] | 178.2[51.8] |
| 7 | Threonine | [M+Na]^+^ | 364.1 | 179.1[29.3] | 178.2[53.7] | 263.1[18.3] |
| 8 | Tryptophan | [M+H]^+^ | 427.1 | 179.1[27.1] | 188.1[17.5] | 205.2[11.8] |
| 9 | Valine | [M+H]^+^ | 340.1 | 179.1[18.6] | 118.2[9.2] | 178.2[47.0] |
|  | ***Non-proteinogenic amino acids*** | | [M+H]^+^ |  |  |  |
| 1 | Citrulline | [M+H]^+^ | 398.1 | 179.1[23.6] | 159.1[14.5] | 178.2[53.8] |
| 2 | GABA | [M+H]^+^ | 326.1 | 179.1[18.6] | 148.1[6.9] | 178.2[45.8] |

Q – quercetin; K – kaempferol

**
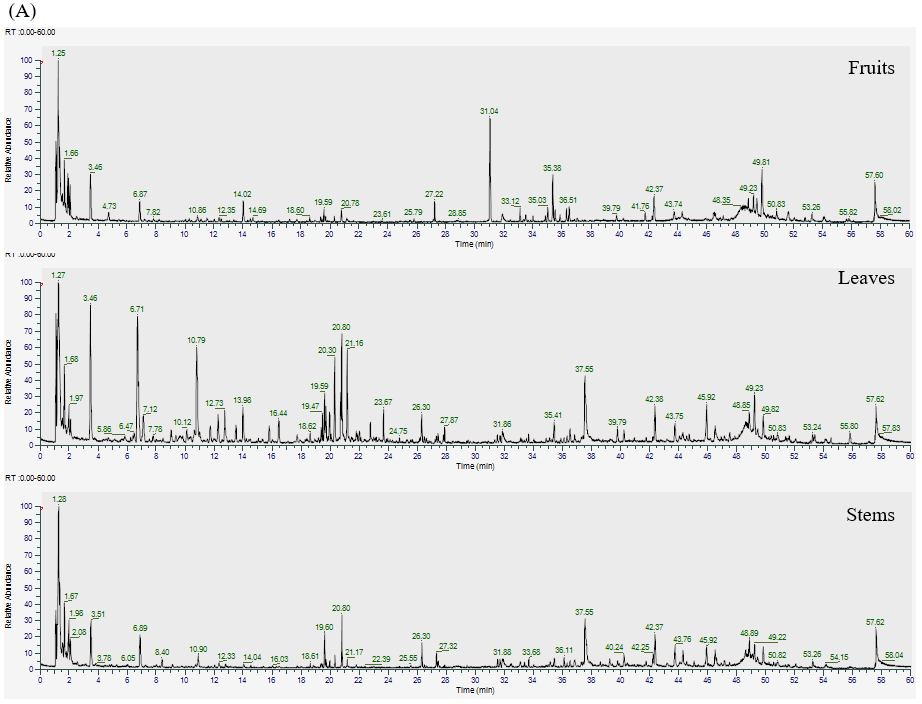
**

**
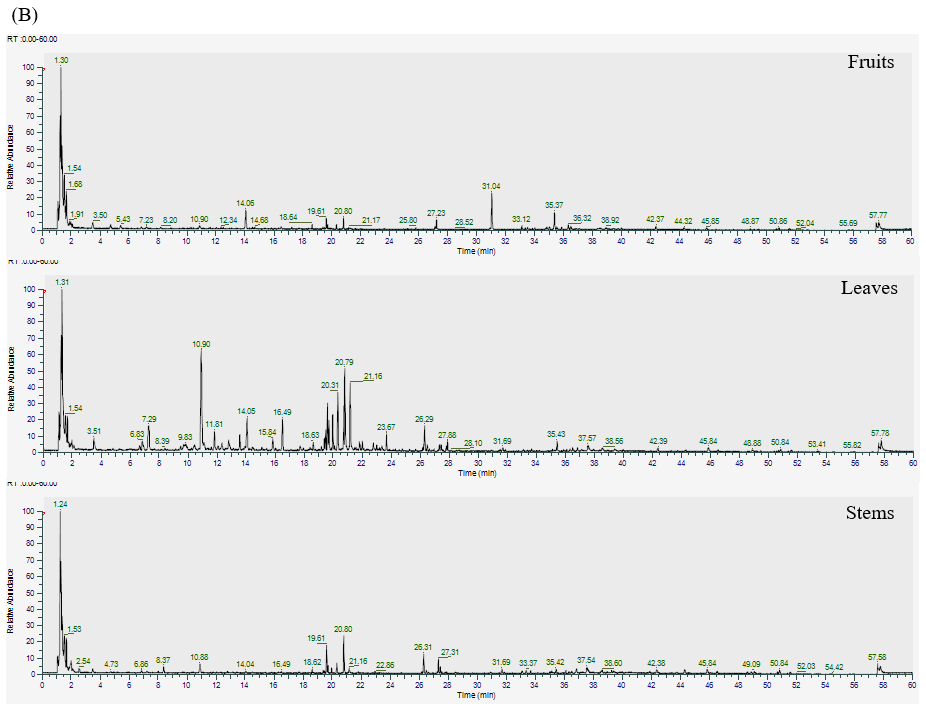
**

**Figure S1. Total ion chromatograms from metabolite profiling in various organs of *Coccinia grandis.***

1. **Positive mode, (B) Negative mode.**

**
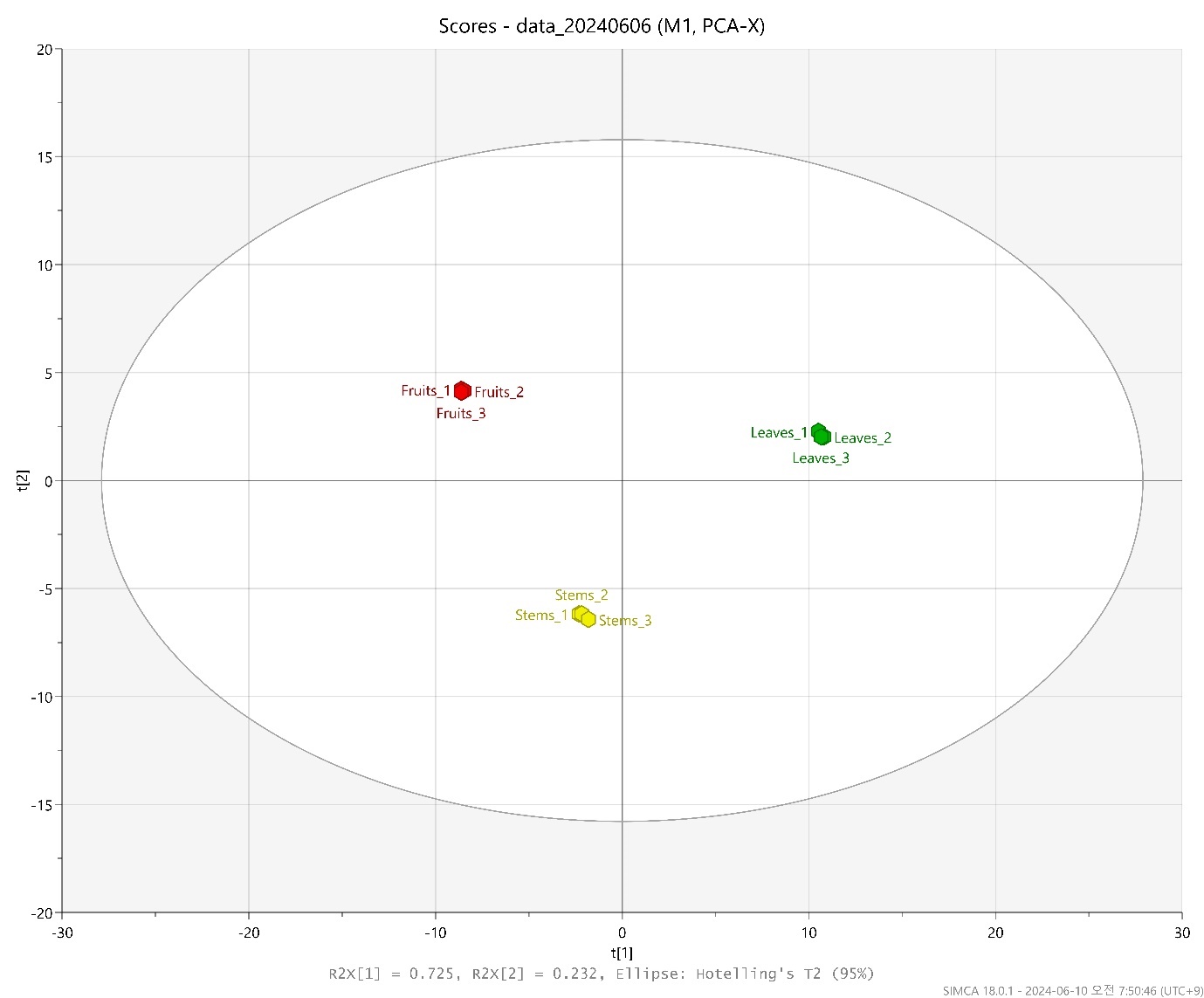
**

**Figure S2. Score plot obtained from metabolite profiling in various organs of *Coccinia grandis.***

**
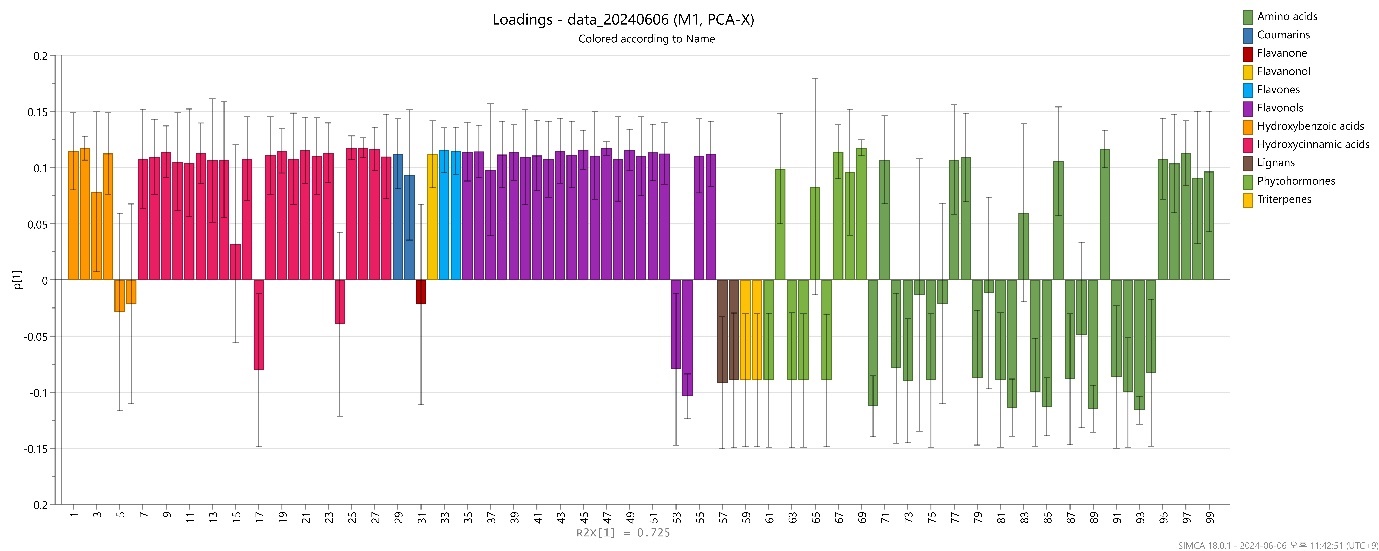
**

**
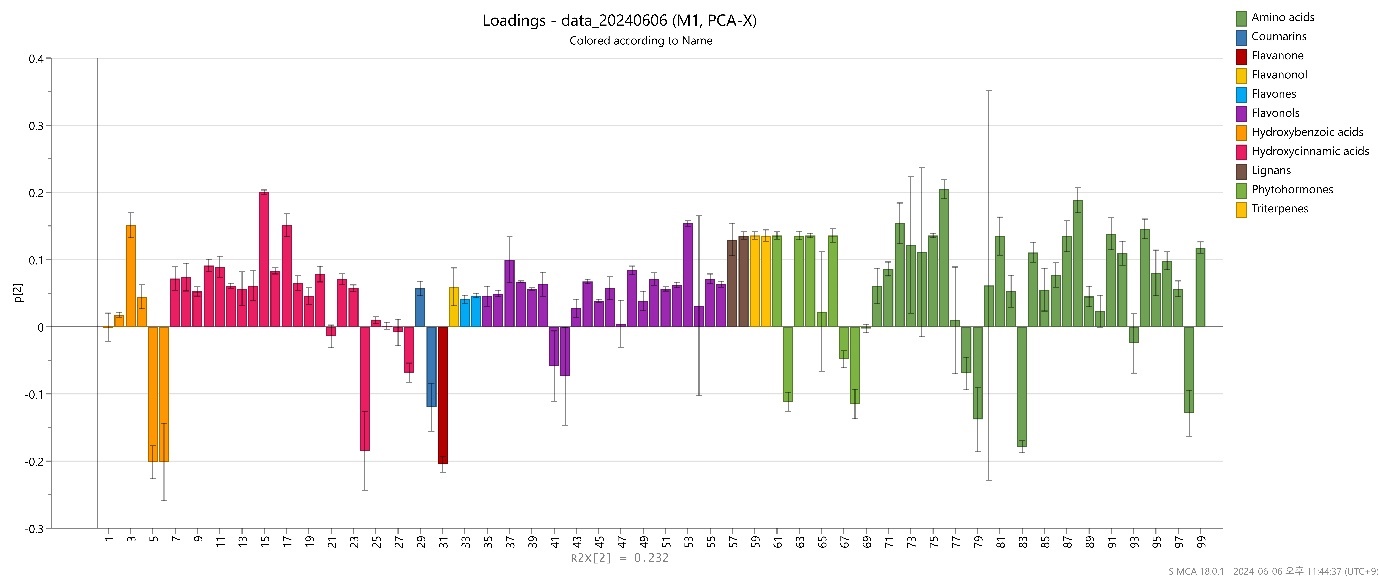
**

**Figure S3. Loading plot obtained from metabolite profiling in various organs of *Coccinia grandis.***

**(A)**

**
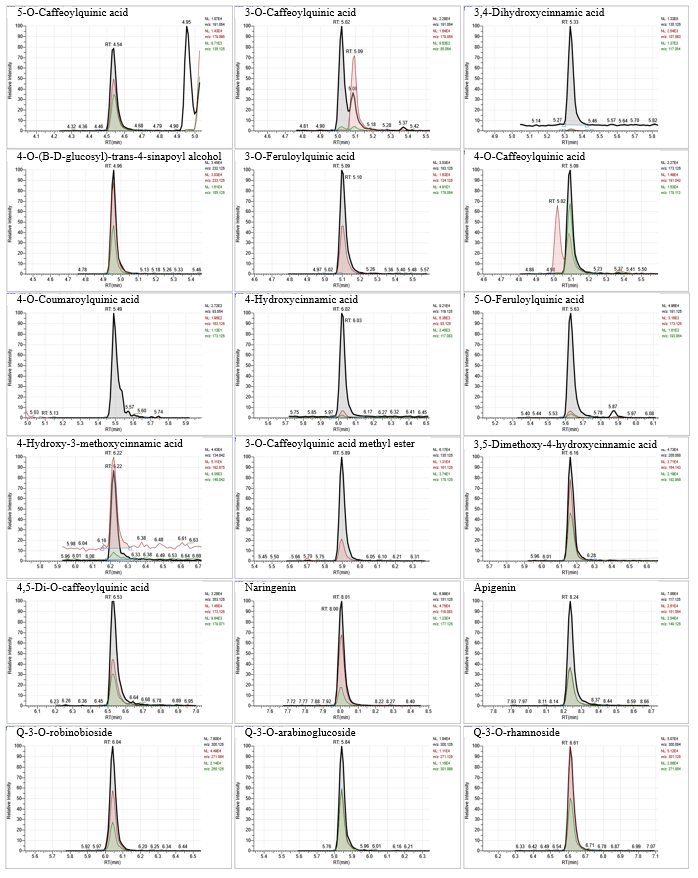
**

(to be continued)

**
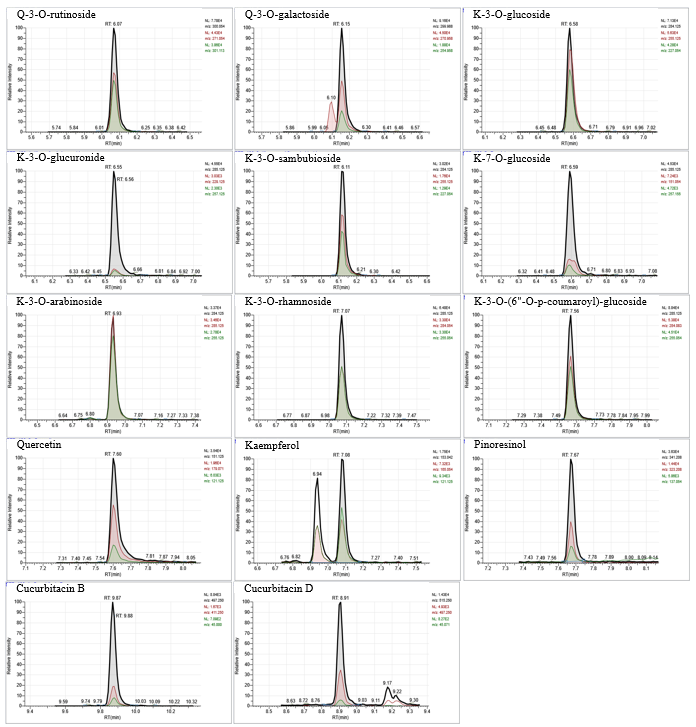
**

**
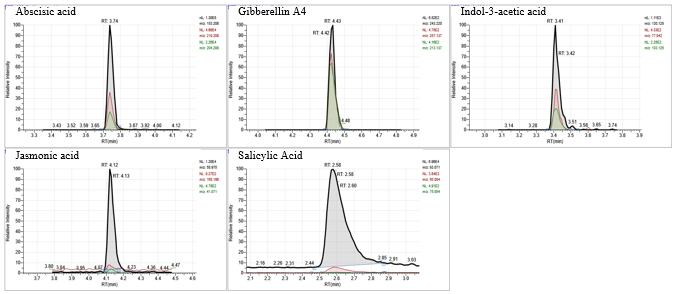
(B)**

(to be continued)


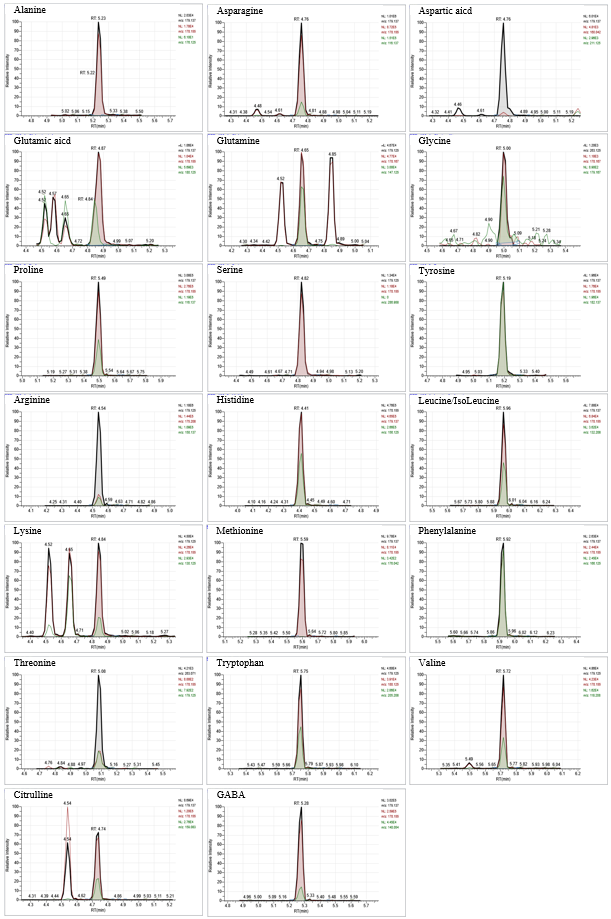
(C)

Figure S4. LC-MS/MS chromatogram of metabolites.

1. Secondary metabolites, (B) Phytohormones, (C) Amino acids.

**(A)**

**
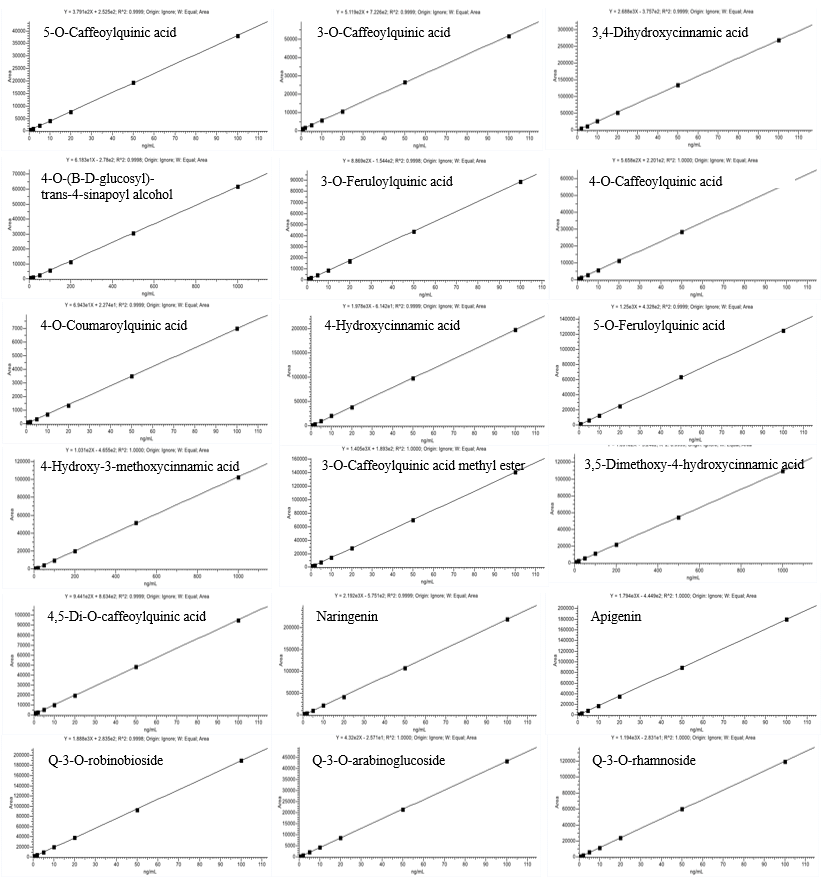
**

(to be continued)

**
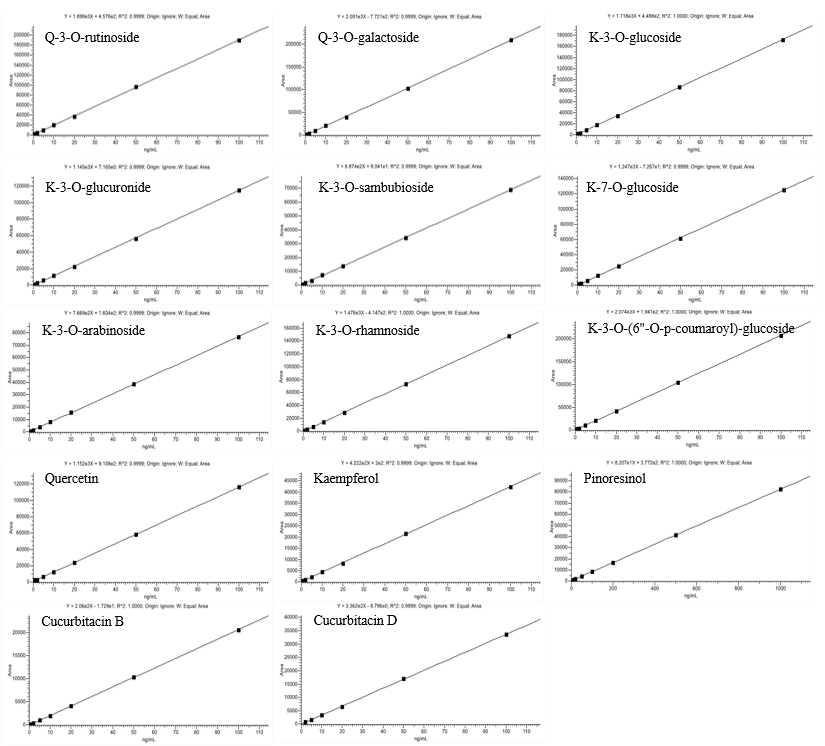
**

**(B)**

**
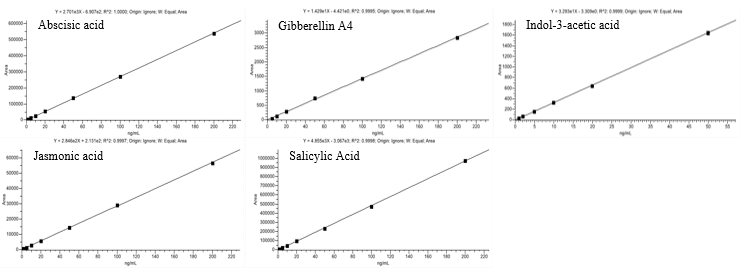
**

(to be continued)

**(C)**

**
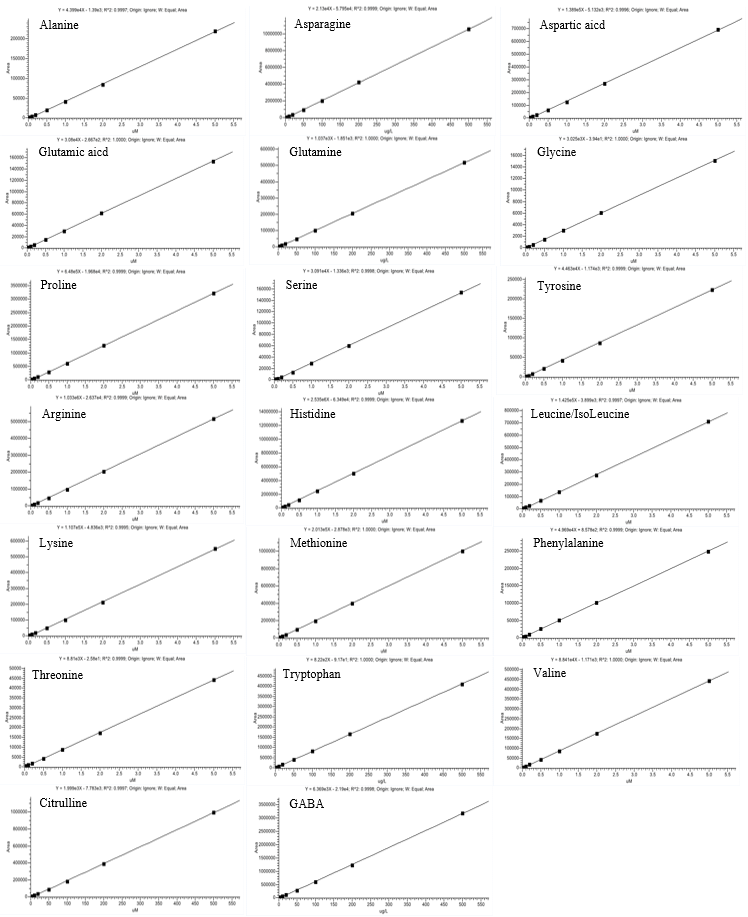
**

**Figure S5. Calibration curves of metabolites.**

1. Secondary metabolites, (B) Phytohormones, (C) Amino acids.
